# Relaxation Therapy and Human Milk Feeding Outcomes: A Systematic Review and Meta-Analysis

**DOI:** 10.1001/jamapediatrics.2024.0814

**Published:** 2024-05-06

**Authors:** Ilana Levene, Nurul Husna Mohd Shukri, Frances O’Brien, Maria A. Quigley, Mary Fewtrell

**Affiliations:** 1 National Perinatal Epidemiology Unit, Nuffield Department of Population Health, University of Oxford, Oxford, United Kingdom; 2 Faculty of Medicine & Health Sciences, Universiti Putra Malaysia, Serdang, Malaysia; 3 Newborn Care, John Radcliffe Hospital, Oxford, United Kingdom; 4 National Perinatal Epidemiology Unit, Nuffield Department of Population Health, University of Oxford, Oxford, United Kingdom; 5 UCL Great Ormond Street Institute of Child Health, London, United Kingdom

## Abstract

**Importance:**

Human milk feeding is a key public health goal to optimize infant and maternal/parental health, but global lactation outcomes do not meet recommended duration and exclusivity. There are connections between lactation and mental health.

**Objective:**

To appraise all available evidence on whether the provision of relaxation interventions to lactating individuals improves lactation and well-being.

**Data Sources:**

Embase, MEDLINE, CINAHL, Allied and Complementary Medicine Database, Web of Science, and the Cochrane Library were searched on September 30, 2023, and topic experts were consulted.

**Study Selection:**

Two independent reviewers screened for eligibility. Inclusion criteria were full-text, peer-reviewed publications with a randomized clinical trial design. Techniques that were entirely physical (eg, massage) were excluded. A total of 7% of initially identified studies met selection criteria.

**Data Extraction and Synthesis:**

Two independent reviewers extracted data and assessed risk of bias with the Cochrane Risk of Bias 2 tool. Fixed-effects meta-analysis and Grading of Recommendations, Assessment, Development, and Evaluations guidelines were used to synthesize and present evidence.

**Main Outcomes and Measures:**

Prespecified primary outcomes were human milk quantity, length and exclusivity of human milk feeding, milk macronutrients/cortisol, and infant growth and behavior.

**Results:**

A total of 16 studies were included with 1871 participants (pooled mean [SD] age for 1656 participants, 29.6 [6.1] years). Interventions were music, guided relaxation, mindfulness, and breathing exercises/muscle relaxation. Provision of relaxation was not associated with a change in human milk protein (mean difference [MD], 0 g/100 mL; 95% CI, 0; 205 participants). Provision of relaxation was associated with an increase in human milk quantity (standardized mean difference [SMD], 0.73; 95% CI, 0.57-0.89; 464 participants), increased infant weight gain in breastfeeding infants (MD, *z* score change = 0.51; 95% CI, 0.30-0.72; 226 participants), and a slight reduction in stress and anxiety (SMD stress score, −0.49; 95% CI, −0.70 to −0.27; 355 participants; SMD anxiety score, −0.45; 95% CI, −0.67 to −0.22; 410 participants).

**Conclusions and Relevance:**

Results of this systematic review and meta-analysis suggest that provision of relaxation was associated with an increase in human milk quantity and infant weight gain and a slight reduction in stress and anxiety. Relaxation interventions can be offered to lactating parents who would like to increase well-being and improve milk supply or, where directly breastfeeding, increase infant weight gain.

## Key Points

**Question:**

What is the association between the provision of a relaxation intervention and lactation outcomes?

**Findings:**

In this systematic review and meta-analysis including 1871 participants, heterogeneous relaxation interventions (including music, meditation, mindfulness, and guided relaxation) were compared with standard care. Results suggest that provision of relaxation was associated with an increase in human milk quantity and infant weight gain and a slight reduction in stress and anxiety.

**Meaning:**

Relaxation interventions can be offered to lactating parents who would like to improve milk supply and increase well-being.

## Teaser

This systematic review and meta-analysis investigates the association between provision of a relaxation intervention and lactation outcomes.

## Introduction

Human milk feeding is an important public health goal with significant economic benefit, but prevalence is low in many countries.^1^ Parents experience increased mental health difficulties after birth.^2^ Inability to meet feeding goals increases the risk of postnatal depression.^3^

Relaxation therapy is an acceptable and low-risk intervention used for many conditions.^4^ It is a complex intervention made up of a variety of techniques.^5^ These include progressive muscle relaxation, meditation, mindfulness, guided visualization, and breathing exercises.^5^ Music is equivalent to formal relaxation techniques in some settings.^6,7^ The common goal for relaxation therapies is to induce a relaxation response characterized by reduced heart rate, respiratory rate, and blood pressure and is associated with a perception of calm and well-being.^5^ Relaxation therapy was identified by a Cochrane review as a promising technique to improve lactation outcomes.^8^

Relaxation therapy could influence lactation via the hormones controlling milk production and release (oxytocin and prolactin) through complex connections with stress hormones.^9,10,11,12^ Relaxation protocols could be the subject of operant conditioning for the milk ejection reflex (also called *letdown*).^13^ Perception of relaxation may influence self-efficacy and behavior, eg, increasing milk expression or feed frequency. If the infant is also exposed to relaxation, there could be direct effects on feeding behaviors and energy use.

This systematic review and meta-analysis aimed to explore the association of relaxation interventions with lactation and well-being. A previous review searched the literature in 2016 and showed limited evidence of effectiveness on milk composition and infant outcomes, with only 5 eligible studies.^14^ The field has evolved significantly since this time, facilitating meta-analysis for the first time.

## Methods

This systematic review and meta-analysis was registered with Prospero (CRD 42021252986) and reported according to the Preferred Reporting Items for Systematic Reviews and Meta-analyses (PRISMA) 2020 reporting guidelines.^15^ Primary outcomes were length and exclusivity of human milk feeding, milk quantity, macronutrients/cortisol, and infant growth and behavior. Secondary outcomes were mental health and other lactation and stress parameters.

Two post hoc amendments to the registered protocol were made to improve generalizability and reduce the potential for bias. These were to include reports in any language and to exclude nonrandomized interventional studies.

### Search Strategy

Embase, MEDLINE, CINAHL Plus, Allied and Complementary Medicine Database, Web of Science, and the Cochrane Library were searched on September 30, 2023. The updated search was limited to articles published in 2016 or later. The search strategy used consistent free-text keywords and Medical Subject Headings terms (eTable 1 in Supplement 1). Topic experts were consulted for any other studies known to them.

### Eligibility Criteria

Articles were eligible for inclusion if they were reported in full text in a peer-reviewed publication with a randomized experimental design and a control group. Data on ethnicity or race were extracted from each study report, in whatever format was used by the original authors. However, most studies did not report these data.

Studies could include any intervention primarily designed to achieve relaxation through mind-stress reduction. Predefined exclusion criteria were manual interventions (eg, massage) and cognitive behavioral therapy. The exclusion of manual therapies is common in systematic review of relaxation interventions.^16,17^

### Study Quality Assessment

Risk of bias was assessed independently by I.L. and N.H.M.S. with the Cochrane Risk of Bias 2 tool (RoB-2).^18^ Consensus discussion was arbitrated by M.F. if required. An overall risk of bias is allocated to each outcome based on the highest risk assessment for any applicable domain.

### Data Extraction

I.L. and N.H.M.S. independently screened abstracts and full-text articles against the eligibility criteria. I.L. extracted data from full-text reports to a standardized form. Trial registration entries, published protocols, and gray literature such as dissertation theses related to the published studies were sought, and authors were contacted for further information.

### Statistical Analysis

If outcomes were reported in several formats, there was a preference for the latest time point measured, standardized outcome measures, and outcomes adjusted for baseline data. A fixed-effects (inverse-variance) technique was used for the meta-analysis, producing forest plots. Categorical outcomes were reexpressed as relative risks. Continuous outcomes were reexpressed as mean differences if data were reported on the same scale or standardized mean differences (SMDs; Cohen method) if not. SMDs of 0.2 to 0.5 are considered a small effect size, and 0.5 to 0.8 are considered a medium effect size.^19^ Where multiple eligible forms of relaxation were separate study arms, these were pooled to compare with the control group. Paired data from crossover studies were derived from *P* values or *t* statistics,^20^ as recommended by Cochrane.^21^ If not possible, the study was included in unpaired format, which is a conservative approach.^20,21^

Publication bias was assessed with use of funnel plots where more than 4 studies contribute to a meta-analysis.^22^ An *I*^2^ statistic of more than 50% was considered to represent substantial statistical heterogeneity. Sensitivity analysis was performed using random effects meta-analysis for primary outcomes.

Grading of Recommendations, Assessment, Development and Evaluation (GRADE)^23^ guidelines were used to assess the overall quality of evidence, using 5 domains to downgrade a baseline assumption of high-quality evidence to moderate, low, or very low.

Stata, version 17 (StataCorp) was used for data analysis, which was performed from September to October 2023. All *P* values were 2-sided, and a *P* value <.05 was considered statistically significant.

## Results

A flowchart for the literature search is presented in eFigure 1 in Supplement 1. Database searches identified 236 individual records (after removal of duplicates), and 1 record was added by a topic expert.^24^ Nineteen of 34 full-text reports assessed were excluded due to ineligible format,^25,26,27,28,29,30,31,32,33^ ineligible outcome,^34,35^ ineligible intervention^36^ and ineligible population or study design.^37,38,39,40,41,42^ Three protocols^43,44,45^ are designated as reports of included studies. The updated search added 15 reports^24,43,44,46,47,48,49,50,51,52,53,54,55,56,57^ of 12 studies to the 4 eligible studies^58,59,60,61^ found in the original search. Of the 16 included studies, there were a total of 1871 participants (pooled mean [SD] age for 1656 participants, 29.6 [6.1] years). One study included in the original systematic review was retrospectively excluded for nonrandomized design.^62^

### Included Studies

Characteristics of included studies are summarized in the Table and in greater detail in eTable 2 and eFigure 2 in Supplement 1. As all included studies used terms such as mother, this term is used in the results of this article. Fourteen studies^24,46,48,49,50,51,53,54,55,56,57,59,60,61^ were parallel group, and 2 studies^47,58^ used a crossover design.

**Table.** Summary of Included Studies

Study	Design	Sample size	Setting	Infant gestation	Intervention (nature)	Intervention (dose)	Original (2016) search?
Ak et al,^58^ 2015	Crossover RCT	30	India, 2012/2013	<34 wk (mean 32.4)	Music (30 min)	Once a day for 4 d	Yes
Chawanpaiboon et al,^56^ 2021	Parallel RCT	620	Thailand, 2018/2019	≥37 wk (mean 38.5)	Music (8 min)	During feeds for up to 2 d	No
Dabas et al,^55^ 2019	Parallel RCT	57	India, 2017	26 to 33 wk	Relaxation practice (30 min)	Once a day for 10 d	No
Dib et al,^48^ 2022	Parallel RCT	72	UK, 2019-2021	34 to 38 wk (mean 36.5)	Relaxation recording (11 min)	At least daily for 2 wk	No
Feher et al,^60^ 1989	Parallel RCT	55	US	<38 wk (mean 31.2)	Relaxation recording (20 min)	Daily for 8-11 d	Yes
Keith et al,^59^ 2012	Parallel RCT	162	US	<38 wk or critically ill (mean 31.9)	Relaxation recording (12 min)	While expressing, for 14 d	Yes
Kittithanesuan et al,^57^ 2017	Parallel RCT	304	Thailand, 2013	≥37 wk (mean 38.5)	Music (11 min)	Once only	No
Massa et al,^50^ 2022	Parallel RCT	70	USA, 2018/2019	24 to 32 wk (mean 30)	Mindfulness meditation app (20 min)	Daily, for nine d	No
Mohd Shukri et al,^44,49^ 2019	Parallel RCT	64	Malaysia, 2014	≥37 wk	Relaxation recording	At least daily for 12 wk	No
Perez-Blasco et al,^61^ 2013	Parallel RCT	26	Spain, 2012	None specified	Mindfulness training (2 h)	Weekly, for eight wk	Yes
Ramesh et al,^24^ 2020	Parallel RCT	81	India	≥37 wk	Music (15 min)	Twice a day for 45 d	No
SefidHaji et al,^54^ 2022	Parallel RCT	100	Iran, 2020	34 to 36 wk (mean 34.8)	Music (30 min)	Once daily for six d	No
Shabnam et al,^53^ 2021	Parallel RCT	70	Iran, 2016/2017	BW 2-2.5 kg (mean 36.8 wk)	Music (5 min)	Three times a day for four wk	No
Varisoglu et al,^51^ 2020	Parallel RCT	44	Turkey, 2017/2018	28 to 34 wk (mean 32)	Music (15 min)	Twice a day for three days	No
Yu et al,^46^ 2023	Parallel RCT	96	China, 2019/2020	34-37 wk (mean 36.1)	Relaxation recording	At least daily for 7 wk	No
Yu et al,^45^ 2019	Crossover RCT	20	China, 2018	None specified	(1) Relaxation recording (2) Music	Once only	No

Abbreviations: BW, birth weight; RCT, randomized clinical trial.

Six studies^46,47,48,49,59,60^ with 469 participants used similar lactation-specific–guided relaxation recordings. Seven studies^24,51,53,54,56,57,58^ with 1249 participants used instrumental music or singing. The remaining studies used yoga breathing exercises and muscle relaxation,^55^ a mindfulness app^50^ and mindfulness training.^61^

Six studies^24,47,49,56,57,61^ included 1115 mothers with term infants or infants with normal birth weight. Ten studies^46,48,50,51,53,54,55,58,59,60^ included 756 mothers with preterm infants or infants with low birth weight (2 of these also included early-term infants^46,48^).

Three trials declared in-kind support from a commercial company—a breast pump manufacturer^47,49^ and the provider of a mindfulness app.^50^

The specific interventions used in 3 of the included studies^46,48,60^ have been provided by the authors as audio files 1 to 5 (English and Chinese language).

### Risk of Bias

**Figure 1.**
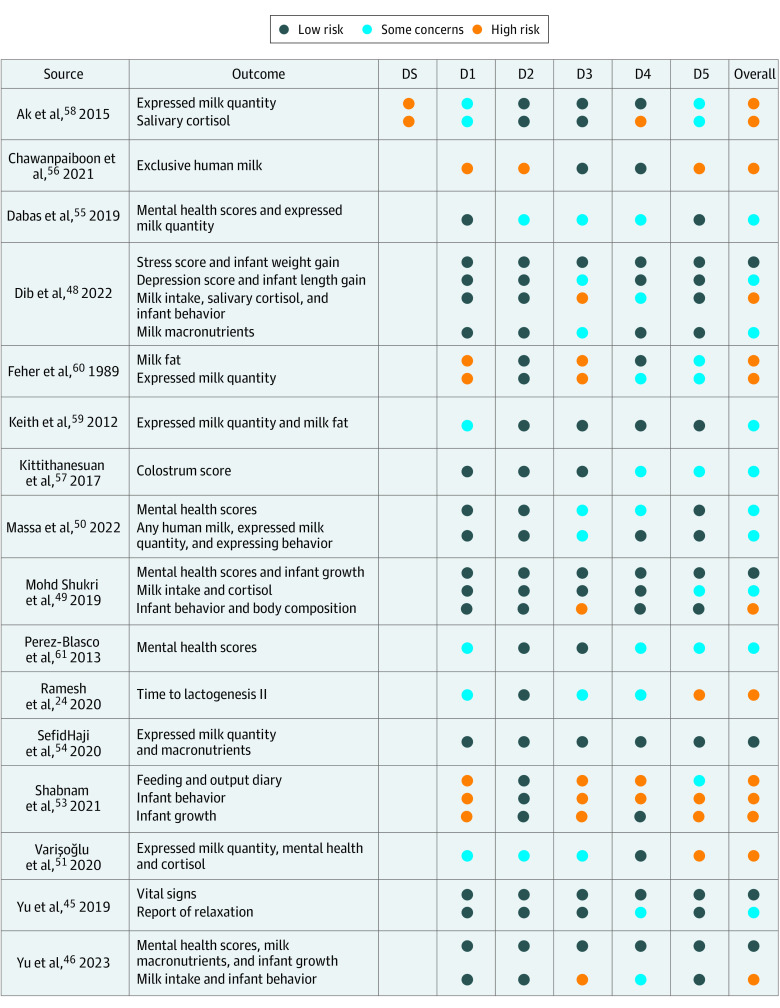
Cochrane Risk of Bias 2 Score for Overall Outcome and Each Domain DS indicates domain S (period and carryover, crossover studies only); D1, domain 1 (randomization); D2, domain 2 (deviations from intended interventions); D3, domain 3 (missing outcome data); D4, domain 4 (outcome measurement); D5, domain 5 (result selection).

Figure 1 shows risk of bias assessments for each outcome using the RoB-2 tool. Nine of 16 studies^24,46,48,49,51,53,56,58,60^ had high risk of bias for at least 1 outcome. Five studies^47,48,49,54^ reported at least 1 outcome with low risk of bias.

### Summaries

**Figure 2.**
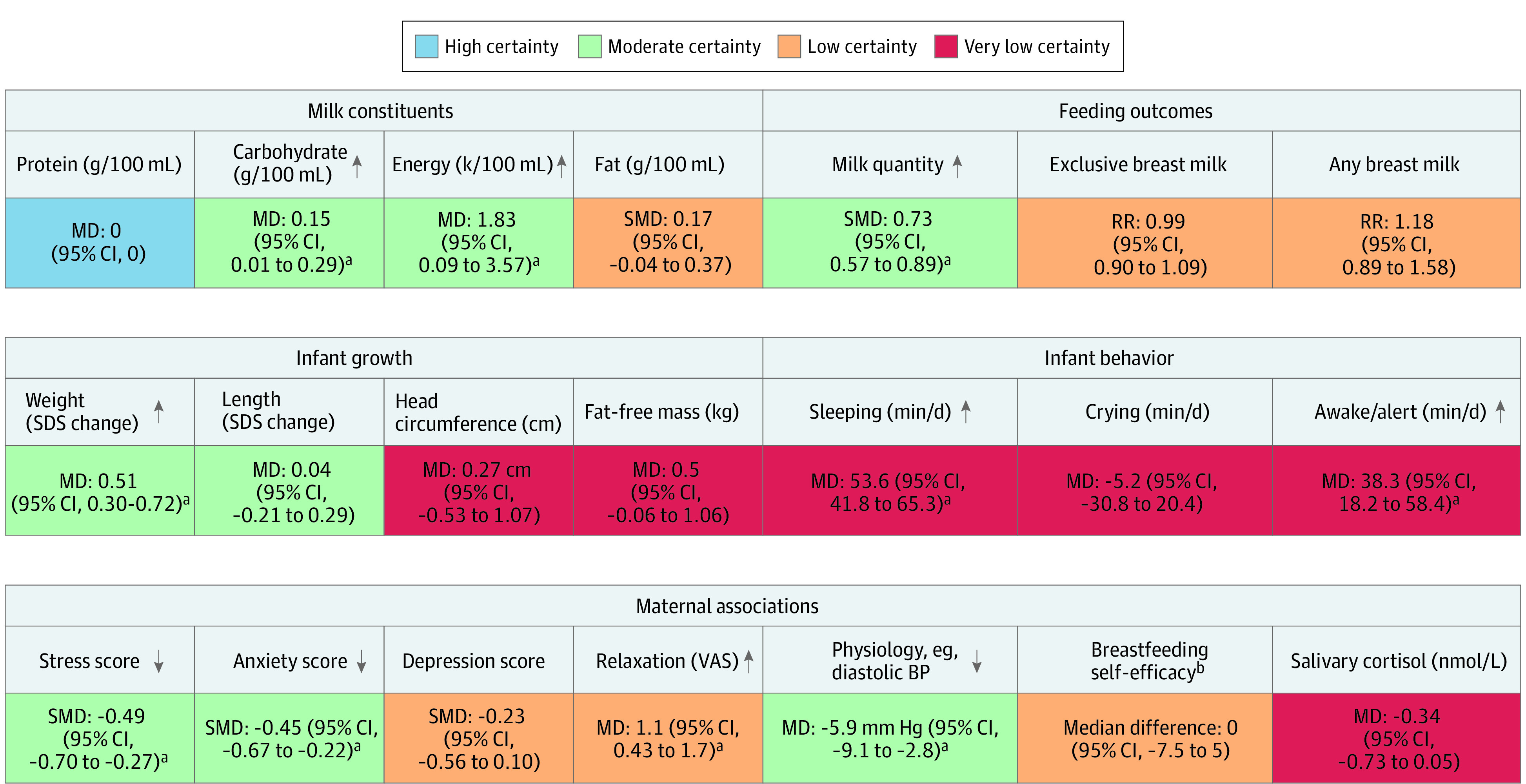
Summary of Meta-Analysis Results BP indicates blood pressure; MD indicates mean difference; RR, relative risk; SDS, standard deviation score; SMD, standardized mean difference; VAS, Visual Assessment Scale. ^a^Indicates significant results (*P* < .05); arrow shows direction of effect. ^b^Measurements were taken using the Breastfeeding Self-Efficacy Scale (short form for the neonatal intensive care unit).

Figure 2 provides an overall summary of the meta-analysis results for each outcome. Due to the proliferation of trials since the outcomes were chosen (before the 2016 search), outcomes with low- and very low–certainty evidence are reported in the eAppendix in Supplement 1, apart from the key outcomes of human milk prevalence. eTable 3 and eTable 4 in Supplement 1 provide further detail on summary outcomes (for high/moderate certainty and low/very low–certainty evidence, respectively), including examples of absolute differences and reasons for downgrading evidence certainty. Data tables and funnel plots are provided in eTable 5 and eFigure 3 in Supplement 1. Forest plots for low- and very low–certainty outcomes are provided in eFigure 4 in Supplement 1.

### Primary Outcomes

There was low-certainty evidence of no difference in any human milk at 1 month of age (relative risk [RR], 1.18; 95% CI, 0.89-1.58, 47 participants) in a single study. This study was classified as some risk of bias due to missing data, with provision of a mindfulness app after very preterm birth.^50^

There was low-certainty evidence of no difference in exclusive human milk at 2 months of age (RR, 0.98; 95% CI, 0.87-1.11; 2 studies, 651 participants) (eFigure 4 in Supplement 1). Two studies contributed to this outcome, providing music for up to 48 hours after birth,^56^ or lactation-specific–guided relaxation for several weeks.^46^ One study^56^ was at high risk of bias due to concerns with the randomization process.

**Figure 3.**
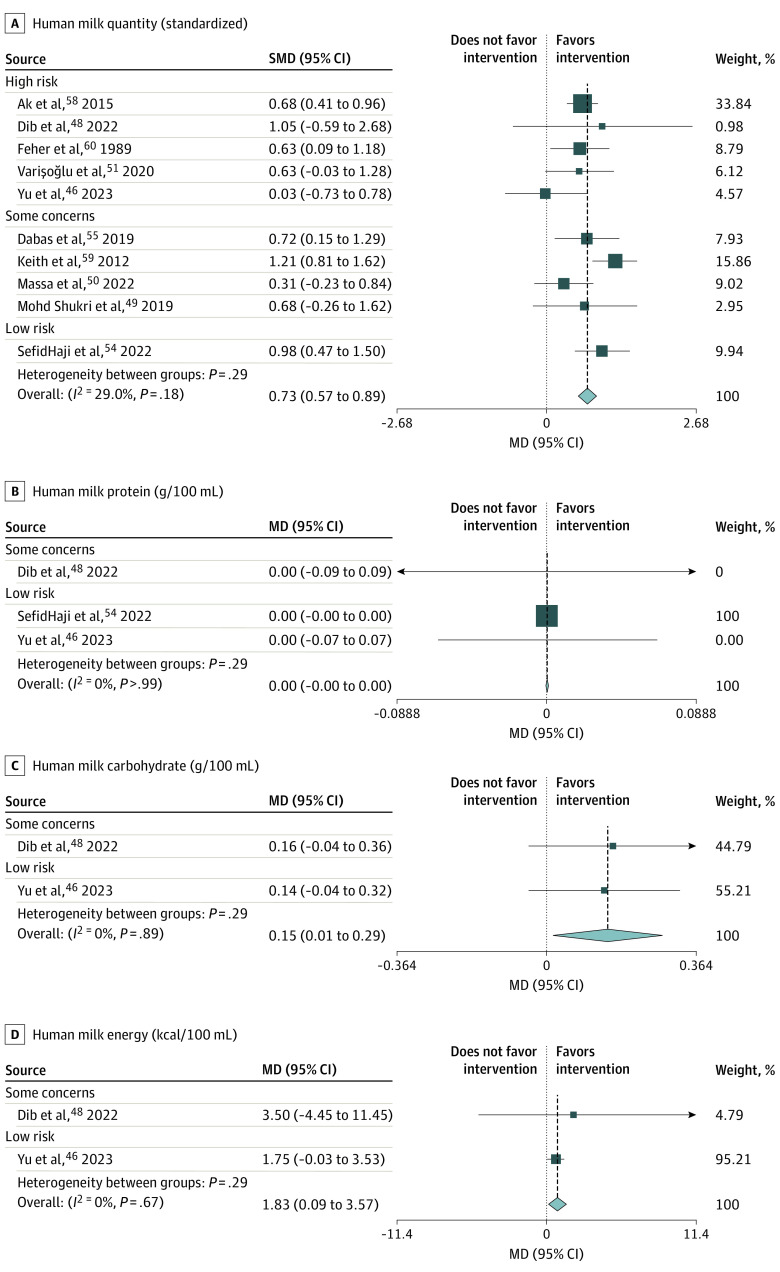
Forest Plots for Human Milk Quantity, Protein, Carbohydrate, and Energy Each forest plot is grouped by risk of bias assessment. MD indicates mean difference; SMD, standardized mean difference.

There was moderate-certainty evidence that relaxation was associated with an increase in milk quantity (SMD, 0.73; 95% CI, 0.57-0.89; *P* < .001; 10 studies, 464 participants) (Figure 3A). This is an increase in milk quantity of 0.73 SDs, a medium effect size.^19^

Ten randomized clinical trials (RCTs) contributed to this outcome, measuring milk quantity expressed in neonatal intensive care unit (NICU) settings^50,51,54,55,58,59,60^ or drunk by a healthy infant at the breast (deuterium isotope or test weighing).^46,48,49^ The studies used lactation-specific–guided relaxation,^46,48,49,59,60^ music,^51,54,58^ breathing exercises,^55^ and a mindfulness app.^50^ Five studies were at high risk of bias for this outcome (due to insufficient washout period in a crossover study,^58^ missing outcome data^46,48,60^ or selection of reporting^51^).

There was high-certainty evidence of no difference in milk protein (mean difference [MD], 0 g/100 mL; 95% CI, 0; 3 studies, 205 participants) (Figure 3B). Three RCTs^46,48,54^ contributed to this outcome, reporting change in milk protein from baseline, spanning an intervention period of 6 days to 8 weeks. One study^48^ was classified as some concerns for risk of bias due to missing data. Although 1 study^54^ dominates the meta-analysis, removing this study produced the same conclusion (MD, 0 g/100 mL; 95% CI, −0.05 to 0.05; 139 participants).

There was moderate-certainty evidence of a small increase in milk carbohydrate (MD, 0.15 g/100 mL; 95% CI, 0.01-0.29 g/100 mL; *P* < .001; 2 studies, 139 participants) (Figure 3C) and milk energy (MD, 1.83 kcal/100 mL; 95% CI, 0.09-3.57 kcal/100 mL; *P* < .001; 2 studies, 139 participants) (Figure 3D). Two RCTs^46,48^ contributed to these 2 outcomes, reporting change in milk carbohydrate and energy from a baseline of week 1 to 3 after birth to a postintervention week 6 to 8. One study^48^ was classified as some concerns for risk of bias due to missing data.

**Figure 4.**
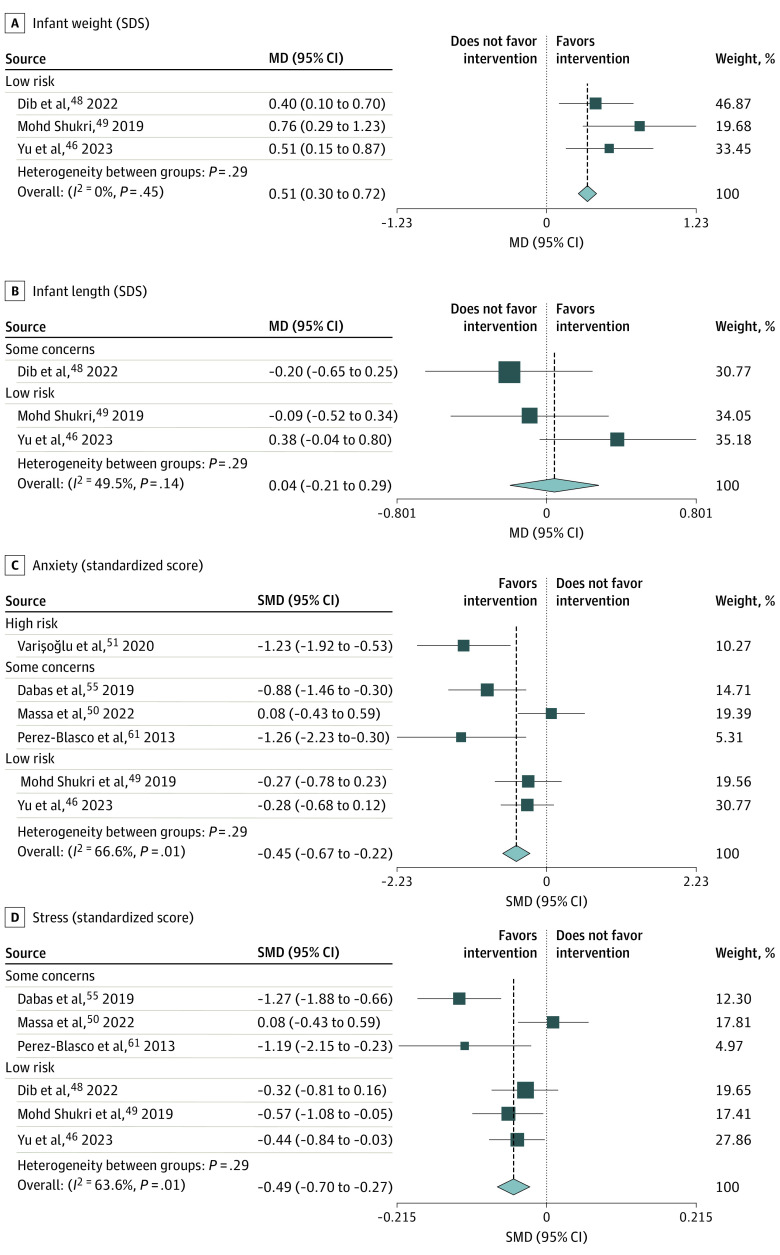
Forest Plots for Infant Weight and Length Gain, Maternal/Parental Anxiety, and Stress Each forest plot is grouped by risk of bias assessment. MD indicates mean difference; SDS, standard deviation score; SMD, standardized mean difference.

There was moderate-certainty evidence of an increase in infant weight, measured as the change in SD score (SDS; MD, *z* score change = 0.51; 95% CI, 0.30-0.72; *P* < .001; 3 studies, 226 participants) (Figure 4A). For comparison, an SDS (also known as *z* score) change of 0.67 is equivalent to moving between the 25th and 50th centiles on a population growth chart.

There was moderate-certainty evidence of no change in infant length (MD, 0.04; 95% CI, −0.21 to 0.29; 3 studies, 214 participants) (Figure 4B). Three RCTs contributed to these 2 growth outcomes, reporting change in SDS between 1 and 2 and 8 and 12 weeks after birth. These studies all used modifications of the same lactation-specific–guided relaxation.^46,48,49^ One study^48^ was classified as some concerns for risk of bias for the outcome of length due to missing data.

A further RCT was not included in these 2 meta-analyses due to nonstandardized outcome data. This study was at high risk of bias due to an imbalance of missing data and concerns over selection of the outcomes reported.^53^ Standardized meta-analysis was possible to combine all 4 studies but heterogeneity would be very high (*I*^2^ = 71% for weight and 88% for length), which was considered unsatisfactory in order to add a study at high risk of bias.

Low- and very low–certainty evidence relating to milk fat, milk cortisol, infant head circumference, body composition, and behavior is described in the eAppendix in Supplement 1.

### Secondary Outcomes

There was moderate-certainty evidence of a reduction in maternal anxiety (SMD, −0.45; 95% CI, −0.67 to −0.22; *P* < .001; 6 studies, 410 participants) (Figure 4C). This is a reduction in anxiety score of 0.45 SDs, a small effect size.^19^ There was substantial statistical heterogeneity (*I*^2^ = 67%).

Six studies^46,49,50,51,55,61^ contributed to this outcome. Interventions were a lactation-specific–guided relaxation,^46,49^ mindfulness app,^50^ instrumental music,^51^ breathing exercises,^55^ and mindfulness training.^61^ One study^51^ was at high risk of bias for this outcome due to inadequate allocation concealment and results selection. Three studies^50,55,61^ were classified as some concern over risk of bias due to the inherent subjectivity of self-reported anxiety in unblinded studies.

There was moderate-certainty evidence of a reduction in maternal stress (SMD, −0.49; 95% CI, −0.70 to −0.27; *P* < .001; 6 studies, 355 participants) (Figure 4D). This is a reduction in stress score of 0.49 SDs, a small effect size.^19^ There was substantial statistical heterogeneity (*I*^2^ = 64%).

Six studies^46,48,49,50,55,61^ contributed to this outcome. Three studies^50,55,61^ were classified as some risk of bias due to the inherent subjectivity of self-reported stress in unblinded studies. One further study^58^ could not be included because of the crossover design and the time point of measurements reported.

There was moderate-certainty evidence for reduction in maternal blood pressure and heart rate and an increase in fingertip temperature in 1 crossover RCT^45^ (eg, diastolic blood pressure MD, −5.9 mm Hg; 95% CI, −9.1 to −2.8 mm Hg; *P* = .001; 20 participants).

Low- and very low–certainty evidence relating to maternal salivary cortisol, perception of relaxation, depression, breastfeeding self-efficacy, breastfeeding frequency, expressing frequency, breastfeeding duration, time to lactogenesis II, and colostrum quantity is described in the eAppendix in Supplement 1.

### Subgroup and Sensitivity Analysis

Human milk yield was the only outcome with sufficient number of studies to explore subgroup outcomes. No significant differences in the pooled effect estimate were seen according to the nature of the relaxation intervention (complexity, timing of onset, dose and whether self-administered or not) or a binary consideration of gestational age at birth (eFigure 5 in Supplement 1).

Sensitivity analysis with random-effects meta-analysis was similar to the fixed-effects results presented.

## Discussion

This systematic review and meta-analysis added 12 new studies and more than 1600 participants to the previous review from 2016, facilitating meta-analysis for the first time. All 5 studies^45,46,48,49,54^ reporting outcomes that were assessed as low risk of bias were published since 2019, which increases the certainty of review conclusions. However, many effect estimates were still assessed as low or very low certainty due to risk of bias and imprecision.

Meta-analysis of current evidence provides high certainty that provision of relaxation was not associated with a change in milk protein content. There is moderate certainty that provision of relaxation was associated with an increase in milk quantity by a moderate, clinically important amount, an increase in infant weight gain by a moderate amount (in the context of direct breastfeeding), a physiological relaxation response in the lactating parent, a reduction in anxiety and stress by a small amount, an increase in milk carbohydrate and energy by a small amount, and no change in infant length.

There is low-certainty evidence that relaxation was associated with an increase in infant sleeping duration, a decrease in immediate milk cortisol, and a perception of relaxation. There is low-certainty evidence that relaxation had no association with prevalence of any or exclusive human milk, milk fat, depression, infant crying duration, frequency of milk expression, and breastfeeding self-efficacy.

Meta-analysis and evidence synthesis is challenging in the context of the very high level of clinical heterogeneity represented within the field of relaxation interventions. The studies were from a wide variety of countries (China, India, Iran, Malaysia, Spain, Thailand, Turkey, UK, and US). The contextual congruence of the intervention is likely to affect adherence; eg, parents report that mindfulness tracks designed for normal birth may be distressing for those experiencing traumatic birth and with sick or preterm infants.^63^ The potential impact of these factors is difficult to assess as each study has a unique cultural context and relevant information about attitudes to relaxation and modifications to the intervention are infrequently reported.

Most studies used lactation-specific–guided relaxation or traditionally relaxing music. Some interventions were delivered or recommended over a period of days, whereas others were over weeks or months. The population was a mixture of parents directly breastfeeding in the community and those expressing milk for sick and preterm babies in the NICU; parents in a neonatal unit are likely to have higher baseline anxiety, distress, and level of lactation challenge.^64,65^ It is surprising but reassuring that in the setting of such clinical heterogeneity that most analyses were not affected by substantial statistical heterogeneity; mental health outcomes were most affected. There was also no suggestion of subgroup differences in the association of relaxation with milk quantity by factors such as dose or infant gestation, although these categorizations were simplistic. This suggests that the outcomes are widely generalizable. No conclusions can currently be drawn on the optimal type of relaxation intervention.

Further research would be helpful for populations with high risk of poor lactation outcomes, particularly those performing time-intensive milk expression routines for infants who are sick, preterm, or with growth concerns who, therefore, need a greater certainty in the impact of investing further time in relaxation interventions. Future studies should aim to integrate objective measures of effect, as well as measures of how participants experienced relaxation interventions, both qualitatively and quantitatively. Objective measures include infant growth and body composition, breast milk composition, independent or standardized assessment of expressed milk volume, and using a deuterium isotope to assess direct breastfeeding milk intake. Using a partial-deception technique for blinding (where neither group knows that the other allocation exists) may be appropriate in settings where participants do not meet each other and where general motivation to engage with relaxation is high.

### Limitations

The key limitation was the quality of available RCTs in this area. More attention should be paid to trial processes such as allocation concealment and prespecified statistical analysis plans to reduce bias in future trials. Crossover studies may not be appropriate in this setting unless parents are at a stable stage of lactation, and the washout period is sufficient. Differential missing data were seen in several trials, with more loss to follow-up in the control arm, particularly for outcomes involving high participant burden such as infant behavior diaries and test weighing.^48,49,53,61^ Attempts for mitigation to this issue should be made for future trials.

## Conclusions

In this systematic review and meta-analysis, the moderate-certainty evidence of an association between relaxation and improvements in infant weight gain, human milk quantity, and mental health suggests that relaxation interventions can be offered to all lactating parents. The lack of major potential harm from relaxation and high acceptability to the general population are further reasons for confidence in this recommendation. Relaxation interventions are easily available for dissemination, particularly the simplest forms using calming music.

## Notes

Supplement 1. **eTable 1.** Search Strategies for Each Database **eTable 2.** Detailed Summary of Included Studies **eTable 3.** Summary of Findings With Examples of Absolute Effect Size (High or Moderate Certainty Outcomes) **eTable 4.** Summary of Findings (Low or Very Low Certainty Outcomes) **eTable 5.** Outcome Data for Individual Meta-Analyses **eFigure 1.** PRISMA Flowchart for a Systematic Review Update **eFigure 2.** Duration of Relaxation Interventions Across Studies **eFigure 3.** Funnel Plots for Individual Meta-Analyses With More Than 4 studies Included **eFigure 4.** Forest Plots for Outcomes With Low and Very Low Certainty **eFigure 5.** Forest Plots for Subgroup Analysis of Human Milk Quantity **eAppendix.** Evidence Summary for Outcomes With Low/Very Low Certainty Not Reported in the Main Text
eReferences

Supplement 2.
Data Sharing Statement.
