# Supplementary material for: Relaxation Therapy and Human Milk Feeding Outcomes: A Systematic Review and Meta-Analysis
Source: JAMA Pediatr. 2024 May 6;178(6):567–76. doi: 10.1001/jamapediatrics.2024.0814 (PMC11074933; doi:10.1001/jamapediatrics.2024.0814)
Supplement: Supplement 1. — eTable 1. Search Strategies for Each Database eTable 2. Detailed Summary of Included Studies eTable 3. Summary of Findings With Examples of Absolute Effect Size (High or Moderate Certainty Outcomes) eTable 4. Summary of Findings (Low or Very Low Certainty Outcomes) eTable 5. Outcome Data for Individual Meta-Analyses eFigure 1. PRISMA Flowchart for a Systematic Review Update eFigure 2. Duration of Relaxation Interventions Across Studies eFigure 3. Funnel Plots for Individual Meta-Analyses With More Than 4 studies Included eFigure 4. Forest Plots for Outcomes With Low and Very Low Certainty eFigure 5. Forest Plots for Subgroup Analysis of Human Milk Quantity eAppendix. Evidence Summary for Outcomes With Low/Very Low Certainty Not Reported in the Main Text eReferences [file jamapediatr-e240814-s001.pdf]

Supplementary Online Content

Levene I, Mohd Shukri NH, O'Brien F, Quigley MA, Fewtrell M. Relaxation therapy and human milk feeding outcomes: a systematic review and meta-analysis. *JAMA Pediatr.* Published online May 6, 2024. doi:10.1001/jamapediatrics.2024.0814

eTable 1. Search Strategies for Each Database

eTable 2. Detailed Summary of Included Studies

eTable 3. Summary of Findings With Examples of Absolute Effect Size (High or Moderate Certainty Outcomes

eTable 4. Summary of Findings (Low or Very Low Certainty Outcomes)

eTable 5. Outcome Data for Individual Meta-Analyses

eFigure 1. PRISMA Flowchart for a Systematic Review Updat

eFigure 2. Duration of Relaxation Interventions Across Studies

eFigure 3. Funnel Plots for Individual Meta-Analyses With More Than 4 studies Included

eFigure 4. Forest Plots for Outcomes With Low and Very Low Certainty

eFigure 5. Forest Plots for Subgroup Analysis of Human Milk Quantity

eAppendix. Evidence Summary for Outcomes With Low/Very Low Certainty Not Reported in the Main Text

eReferences

This supplementary material has been provided by the authors to give readers additional information about their work.

eTable 1: Search Strategies for each database

AMED:

**1.**	exp breast milk/
**2.**	exp breast feeding/
**3.**	exp lactation/ or exp lactation disorder/
**4.**	breastmilk.mp. [mp=abstract, heading words, title]
**5.**	"human milk".mp. [mp=abstract, heading words, title]
**6.**	breastfed.mp. [mp=abstract, heading words, title]
**7.**	breastfeeding.mp. [mp=abstract, heading words, title]
**8.**	"breast feeding".mp. [mp=abstract, heading words, title]
**9.**	"breast milk".mp. [mp=abstract, heading words, title]
**10.**	lactation.mp. [mp=abstract, heading words, title]
**11.**	exp relaxation training/
**12.**	exp guided imagery/ or exp imagery/
**13.**	exp meditation/
**14.**	exp music therapy/
**15.**	exp Milk, Human/
**16.**	exp Relaxation Therapy/
**17.**	exp Imagery, Psychotherapy/
**18.**	exp Relaxation/ or exp Muscle Relaxation/
**19.**	"relaxation therapy".mp. [mp=abstract, heading words, title]
**20.**	meditation.mp. [mp=abstract, heading words, title]
**21.**	"guided imagery".mp. [mp=abstract, heading words, title]
**22.**	"verbal protocol".mp. [mp=abstract, heading words, title]
**23.**	"music therapy".mp. [mp=abstract, heading words, title]
**24.**	1 or 2 or 3 or 4 or 5 or 6 or 7 or 8 or 9 or 10 or 15
**25.**	11 or 12 or 13 or 14 or 16 or 17 or 18 or 19 or 20 or 21 or 22 or 23
**26.**	24 and 25
**27.**	exp music/
**28.**	25 or 27
**29.**	24 and 28

© 2024 Levene I et al. *JAMA Pediatrics.*

MEDLINE:

**1.**	exp breast milk/
**2.**	exp breast feeding/
**3.**	exp lactation/ or exp lactation disorder/
**4.**	breastmilk.mp. [mp=title, abstract, original title, name of substance word, subject heading word, floating sub-heading word, keyword heading word]
**5.**	"human milk".mp. [mp=title, abstract, original title, name of substance word, subject heading word, floating sub-heading word, keyword heading word]
**6.**	breastfed.mp. [mp=title, abstract, original title, name of substance word, subject heading word, floating sub-heading word, keyword heading word]
**7.**	breastfeeding.mp. [mp=title, abstract, original title, name of substance word, subject heading word, floating sub-heading word, keyword heading word]
**8.**	"breast feeding".mp. [mp=title, abstract, original title, name of substance word, subject heading word, floating sub-heading word, keyword heading word]
**9.**	"breast milk".mp. [mp=title, abstract, original title, name of substance word, subject heading word, floating sub-heading word, keyword heading word]
**10.**	lactation.mp. [mp=title, abstract, original title, name of substance word, subject heading word, floating sub-heading word, keyword heading word]
**11.**	exp relaxation training/
**12.**	exp guided imagery/ or exp imagery/
**13.**	exp meditation/
**14.**	exp music therapy/
**15.**	exp Milk, Human/
**16.**	exp Relaxation Therapy/
**17.**	exp Imagery, Psychotherapy/
**18.**	exp Relaxation/ or exp Muscle Relaxation/
**19.**	"relaxation therapy".mp. [mp=title, abstract, original title, name of substance word, subject heading word, floating sub-heading word, keyword heading word]
**20.**	meditation.mp. [mp=title, abstract, original title, name of substance word, subject heading word, floating sub-heading word, keyword heading word]
**21.**	"guided imagery".mp. [mp=title, abstract, original title, name of substance word, subject heading word, floating sub-heading word, keyword heading word]
**22.**	"verbal protocol".mp. [mp=title, abstract, original title, name of substance word, subject heading word, floating sub-heading word, keyword heading word]
**23.**	"music therapy".mp. [mp=title, abstract, original title, name of substance word, subject heading word, floating sub-heading word, keyword heading word]
**24.**	1 or 2 or 3 or 4 or 5 or 6 or 7 or 8 or 9 or 10 or 15
**25.**	11 or 12 or 13 or 14 or 16 or 17 or 18 or 19 or 20 or 21 or 22 or 23
**26.**	24 and 25
**27.**	exp music/
**28.**	25 or 27
**29.**	24 and 28

© 2024 Levene I et al. *JAMA Pediatrics.*

Embase:

**1.**	exp breast milk/
**2.**	exp breast feeding/
**3.**	exp lactation/ or exp lactation disorder/
**4.**	breastmilk.mp. [mp=title, abstract, heading word, drug trade name, original title, device manufacturer, drug manufacturer, device trade name, keyword, floating subheading word, candidate term word]
**5.**	"human milk".mp. [mp=title, abstract, heading word, drug trade name, original title, device manufacturer, drug manufacturer, device trade name, keyword, floating subheading word, candidate term word]
**6.**	breastfed.mp. [mp=title, abstract, heading word, drug trade name, original title, device manufacturer, drug manufacturer, device trade name, keyword, floating subheading word, candidate term word]
**7.**	breastfeeding.mp. [mp=title, abstract, heading word, drug trade name, original title, device manufacturer, drug manufacturer, device trade name, keyword, floating subheading word, candidate term word]
**8.**	"breast feeding".mp. [mp=title, abstract, heading word, drug trade name, original title, device manufacturer, drug manufacturer, device trade name, keyword, floating subheading word, candidate term word]
**9.**	"breast milk".mp. [mp=title, abstract, heading word, drug trade name, original title, device manufacturer, drug manufacturer, device trade name, keyword, floating subheading word, candidate term word]
**10.**	lactation.mp. [mp=title, abstract, heading word, drug trade name, original title, device manufacturer, drug manufacturer, device trade name, keyword, floating subheading word, candidate term word]
**11.**	exp relaxation training/
**12.**	exp guided imagery/ or exp imagery/
**13.**	exp meditation/
**14.**	exp music therapy/
**15.**	exp Milk, Human/
**16.**	exp Relaxation Therapy/
**17.**	exp Imagery, Psychotherapy/
**18.**	exp Relaxation/ or exp Muscle Relaxation/
**19.**	"relaxation therapy".mp. [mp=title, abstract, heading word, drug trade name, original title, device manufacturer, drug manufacturer, device trade name, keyword, floating subheading word, candidate term word]
**20.**	meditation.mp. [mp=title, abstract, heading word, drug trade name, original title, device manufacturer, drug manufacturer, device trade name, keyword, floating subheading word, candidate term word]
**21.**	"guided imagery".mp. [mp=title, abstract, heading word, drug trade name, original title, device manufacturer, drug manufacturer, device trade name, keyword, floating subheading word, candidate term word]
**22.**	"verbal protocol".mp. [mp=title, abstract, heading word, drug trade name, original title, device manufacturer, drug manufacturer, device trade name, keyword, floating subheading word, candidate term word]
**23.**	"music therapy".mp. [mp=title, abstract, heading word, drug trade name, original title, device manufacturer, drug manufacturer, device trade name, keyword, floating subheading word, candidate term word]
**24.**	1 or 2 or 3 or 4 or 5 or 6 or 7 or 8 or 9 or 10 or 15
**25.**	11 or 12 or 13 or 14 or 16 or 17 or 18 or 19 or 20 or 21 or 22 or 23
**26.**	24 and 25
**27.**	exp music/
**28.**	25 or 27
**29.**	24 and 28

© 2024 Levene I et al. *JAMA Pediatrics.*

CINAHL:

**S1.**	(MH "Breast Feeding+")
**S2.**	(MH "Lactation") OR (MH "Lactation Disorders+")
**S3.**	(MH "Milk, Human")
**S4.**	"human milk" OR breastmilk OR "breast milk" OR breastfeeding OR "breast feeding" OR breastfed OR lactation
**S5.**	S1 OR S2 OR S3 OR S4
**S6.**	(MH "Relaxation") OR (MH "Relaxation Techniques+") OR (MH "Muscle Relaxation") OR (MH "Progressive Muscle Relaxation (Iowa NIC)")
**S7.**	(MH "Guided Imagery")
**S8.**	(MH "Simple Relaxation Therapy (Iowa NIC)")
**S9.**	(MH "Meditation") OR (MH "Meditation (Iowa NIC)")
**S10.**	(MH "Music Therapy") OR (MH "Music Therapy (Iowa NIC)")
**S11.**	"relaxation therapy" OR "music therapy" OR meditation OR "guided imagery" OR "verbal protocol"
**S12.**	(MH "Music")
**S13.**	S6 OR S7 OR S8 OR S9 OR S10 OR S11 OR S12
**S14.**	S5 AND S13

Web Of Science:

**#1.**	ALL=("relaxation therapy" OR "relaxation technique" OR music OR imagery OR meditation OR "verbal protocol" OR "muscle relaxation")
**#2.**	ALL=("breast milk" OR breastmilk OR lactation OR "human milk" OR breastfeeding OR "breast feeding" OR breastfed)
**#3.**	#1 AND #2

© 2024 Levene I et al. *JAMA Pediatrics.*

eTable 2: Detailed summary of included studies

Study ID and design	Setting	Key inclusion criteria	Key baseline characteristics	Intervention and control conditions	Outcomes included
**Ak 2015**[1]<br><br>**Crossover RCT**	N = 30<br><br>University Hospital NICU, India, 2012-2013	Infants:<br>Born <34 weeks' PMA, admitted to NICU	Mean age 24 years.<br><br>45% had caesarean birth<br><br>Infants: Mean PMA 32.4 weeks' (provided by authors on request)	Intervention: 30 minutes of Indian flute music (headphones, supervised)<br><br>Instruction: Listen for 30 minutes once a day for four days (randomised to listening at the morning or afternoon expressing session each day)<br><br>Control: same conditions without music	Milk quantity: milk expressed during 15 minute expressing session at 11am or 4pm, each day for four days<br><br>Salivary cortisol: samples taken before and after the intervention expressing session on the fourth day of the study
**Chawanpaiboon et al 2021**[2]<br><br>**Parallel group RCT**	N = 620<br><br>University Hospital postnatal ward, Thailand, 2018-2019	Single pregnancy, elective caesarean birth<br><br>Infant: Born at ≥37 weeks' PMA, not admitted to NICU	Mean age 31-34 years, most had a university education<br><br>Infants:<br>Mean PMA 38 - 39 weeks'	Intervention: Eight minutes of a calming Thai song about breastfeeding (headphones).<br><br>Two intervention arms were a) listening solely in the delivery theatre, or b) listening in the delivery theatre and during breastfeeding for the next two days<br><br>Control: standard care	Exclusive breastfeeding: Telephone interview at 7 days; 14 days; 1 month, 2 months, 3 months and 6 months of the infant's life<br><br>Note, the two intervention arms have been pooled meta-analysis, producing a 2:1 allocation ratio
**Dabas et al 2019**[3]<br><br>**Parallel group RCT**	N = 57<br><br>University Hospital NICU, India, 2017	Single pregnancy, 18-45 years<br><br>Infant:<br>Born at 26 – 33 weeks' PMA, admitted to NICU	Mean age 29-30 years, most had graduate education or above<br><br>Infants: 28-40% had BW <1kg	Intervention: 30 minute relaxation practice including yoga breathing exercises and progressive muscle relaxation<br><br>Instruction: Guided practice for 30 minutes each day, for 10 consecutive days<br><br>Control: Standard care	Parental Stress Scale for the Neonatal Intensive Care Unit (score of 1-5 on each of three dimensions): self-report at day 10<br><br>Perinatal Anxiety Screening Scale (31 items and score 0-93): self-report at day 10<br><br>Milk quantity: milk expressed with electric pump until emptying, divided by frequency of expression. 24 hour period at day 10

© 2024 Levene I et al. *JAMA Pediatrics.*

Study ID and design	Setting	Key inclusion criteria	Key baseline characteristics	Intervention and control conditions	Outcomes included
**Dib et al 2022[4,5]**<br><br>**Parallel group RCT**	N = 72<br><br>Urban capital, UK, 2019-2021	Intention to breastfeed for > 6 weeks, non-smoker, English speaking<br><br>Infant: singletons born at 34 to 38 weeks' PMA	Mean age 33 years. 68% primiparous<br>73% Bachelor's degree or higher<br>55% White, 16% Black, 17% Asian<br>49% caesarean birth<br><br>Infants: mean PMA 36.5 weeks'	Intervention: lactation focused recording (including muscle relaxation and guided imagery). Modified version of a commercial recording<br><br>Instruction: listen while breastfeeding, at least daily for 2 weeks<br><br>Control: standard care with breastfeeding support.<br><br>Neither group was aware that they were in a randomised trial or that other participants were receiving/not receiving relaxation.	Outcomes measured at two (baseline) and six weeks after birth<br><br>Milk intake: test weighing infant before and after each feed<br><br>Infant diary: three-day diary record, recorded at fifteen minute epochs<br><br>Infant weight: measured on a digital scale. Standard deviation scores using Intergrowth-21 growth charts<br><br>Perceived Stress Scale (14 items and 0-40 score).<br>Edinburgh Postnatal Depression Score (10 items and 0-30 score)<br><br>Salivary cortisol: passive drool method at midmorning<br><br>Breastmilk constituents: express milk from when the breast is full to empty at midmorning and at least 2 hours from a previous feed. Sample mixed
**Feher et al 1989[6]**<br><br>**Parallel group RCT**	N = 55<br><br>University and urban private NICUs, USA	Three to five days postpartum<br><br>Infant:<br>Born preterm and expected to be in the NICU for at least 10 days	Mean age 25 to 27 years<br><br>43-68% primiparous<br><br>Infants:<br>Mean PMA 31-32 weeks'	Intervention: 20 minute lactation focused recording (including progressive muscle relaxation and guided imagery)<br><br>Instruction: listen once daily until expressing assessment<br><br>Control: standard care	Milk quantity: single expressing session of undefined length targeted at one week after enrolment (occurred at 4-13 days of the study, mean 8 days of the study)<br><br>Milk fat: sample expressed a minimum of two hours after previous expression, targeted at one week after enrolment

© 2024 Levene I et al. *JAMA Pediatrics.*

Study ID and design	Setting	Key inclusion criteria	Key baseline characteristics	Intervention and control conditions	Outcomes included
**Keith et al 2012**[7]<br><br>**Parallel group RCT**	N = 162<br><br>NICU, Urban USA	Non-smokers<br><br>Infant: Born <38 weeks' PMA or critically ill and admitted to NICU	Mean age 25 to 29 years<br><br>Twin pregnancies 13-16%<br><br>Infants:<br>Mean PMA 31-33 weeks'	Intervention 1: 12 minute lactation focused recording (including muscle relaxation and guided imagery). Modified version of a commercial recording.<br><br>Intervention 2: additional music behind the voice recording described in intervention 1<br><br>Instruction: listen while expressing milk as often as possible, for 14 days. Given mp3 player<br><br>Control: standard care. Note one further intervention arm is not included in this review	Milk quantity: expressing diary kept with participant measurement of milk volume from day 1 to 14 of study<br><br>Milk fat: 1ml sample of composite milk collected daily around noon from day 1 to 14 of study<br><br>Note, the two intervention arms included have been pooled for meta-analysis, producing a 2:1 allocation ratio
**Kittithanesuan et al 2017**[8]<br><br>**Parallel group RCT**	N = 304<br><br>Provincial hospital, Thailand, 2013	Vaginal birth<br><br>Infant:<br>Born at ≥37 weeks' PMA	Mean age 25-26 years, most had junior high school level education<br><br>49% previously breastfed<br><br>Infants:<br>Mean PMA 38.5 weeks'	Intervention: 11 minutes of a calming Thai song about breastfeeding<br><br>Instruction: played in the delivery room once<br><br>Control: Standard care	Colostrum score: nurses in the delivery room performed a single compression of each breast within the first hour of life and after the first feed, before 2 hours of life. Scored 0-3 according to the amount of colostrum expressed
**Massa et al 2022**[9]<br><br>**Parallel group RCT**	N = 70<br><br>University Hospital NICU, USA, 2018-2019	Planned to provide milk for at least a month<br><br>Infant: Born at 24 to 32 weeks' PMA, less than 48 hours old	Mean age 27 to 29 years<br><br>52% Black<br>41-48% nulliparous<br>16 – 21% multiple birth<br>58 – 66% caesarean birth<br><br>Infants:<br>Mean PMA 30 weeks'	Intervention: free access to a commercial mindfulness-based meditation app for postpartum period<br><br>Instruction: Use daily for 20 minutes, signposted to tracks related to lactation and postpartum recovery<br><br>Control: Standard care (received access to the app after study end)	24-hour milk yield, expressing frequency, expressing method: day 9 of the infant's life, participant diary<br><br>Between day 10 & 16 of the infant's life: Edinburgh Postnatal Depression Scale; Spielberger State-Trait Anxiety Index; Perceived Stress Scale: NICU; Breastfeeding Self-Efficacy Scale short form for NICU<br><br>Any breastmilk feeding: telephone interview at 28 days of infant's life

© 2024 Levene I et al. *JAMA Pediatrics.*

Study ID and design	Setting	Key inclusion criteria	Key baseline characteristics	Intervention and control conditions	Outcomes included
**Mohd Shukri 2019**[10,11]<br><br>**Parallel RCT**	N = 64<br><br>Urban Malaysia, 2014	Primiparous, non-smoker, exclusive breastfeeding at 2 weeks after birth<br><br>Infant:<br>Born at ≥37 weeks' PMA and BW ≥2.5kg	Most were 26-30 years old with a Bachelor's degree and a goal to breastfeed for more than 19 months<br><br>75% had a vaginal birth	Intervention: lactation focused recording (including muscle relaxation and guided imagery), translated into Malay with minor amendments (English and Malay versions available to participants). Modified from a commercial recording<br><br>Instruction: listen to the recording while breastfeeding or expressing at least daily from 2 to 14 weeks of age<br><br>Control: standard care, including breastfeeding education material from the researcher.<br><br>Neither group was aware that they were in a randomised trial or that other participants were receiving/not receiving relaxation.	Outcomes recorded at two and six weeks after birth unless otherwise mentioned<br><br>Cohen's Perceived Stress Score, Edinburgh Postnatal Depression Scale and Beck Anxiety Inventory (21 items and score 0-63): self-report at two, six and 12-14 weeks after birth<br><br>Breastmilk intake: deuterium isotope to mother technique at two and 12 weeks<br><br>Breastmilk cortisol: samples taken before and after listening to the intervention (or equivalent time), in the morning<br><br>Salivary cortisol: samples taken before and after listening to the intervention (or equivalent), in the morning<br><br>Infant diary: three-day diary record, recorded at five minute epochs<br><br>Infant weight: measured at two, six and 12 weeks. Standard deviation scores - World Health Organisation 2006 growth charts<br><br>Infant body composition: deuterium to infant technique at 12 weeks
**Perez-Blasco et al 2013**[12]<br><br>**Parallel group RCT**	N = 26<br><br>Urban Spain, 2012	Lactating	Mean age 34 years<br><br>57% primiparous<br><br>Infants:<br>Mean age 10.8 months	Intervention: two hour mindfulness training with babies present. Each session included several 10 minute meditations<br><br>Instruction: attend weekly, for eight weeks<br><br>Control: standard care (mindfulness instruction after study end)	Depression, Anxiety and Stress Scale (each of the three subscales is 7 item, score 0-21): self-report at baseline and 11 weeks into the study (three weeks after the intervention was complete)

© 2024 Levene I et al. *JAMA Pediatrics.*

Study ID and design	Setting	Key inclusion criteria	Key baseline characteristics	Intervention and control conditions	Outcomes included
**Ramesh et al 2020**[13]<br><br>**Parallel group RCT**	N = 81<br><br>University Hospital postnatal ward, India<br><br>Year not reported	Primiparous, 19-35 years old<br><br>Infant: Born at ≥37 weeks' PMA	Most were 21-25 years old and had graduated from high school	Intervention: 15 minutes of Indian classical flute music and encouraged to sing lullabies in the regional language for 15 minutes<br><br>Instruction: Listen/sing twice a day for 45 days<br><br>Control: Standard care	Timing of lactogenesis II (milk 'coming in'): UNICEF checklist each day for four days
**SefidHaji et al 2022**[14]<br><br>**Parallel group RCT**	N = 100<br><br>Regional hospital, Iran, 2020	Primiparous, intention to breastfeed, no use of galactagogues or drug abuse, no serious health issues<br><br>Infant: between 34 and 36 weeks' PMA	Mean age 29 – 30 years.<br><br>64 – 91% moderate economic status<br><br>Weight 61 - 62kg<br><br>Infants: mean PMA 34.8 weeks	Intervention: 30 minutes of traditional Iranian lullabies (headphones, supervised)<br><br>Instruction: Around noon, once daily for six days<br><br>Control: standard care with breastfeeding instruction. Note, a second intervention arm was not included in the review	Milk quantity: volume expressed in 15 minutes with double electric pump on day one and six of study (for intervention group - half way through the intervention period)<br><br>Breastmilk constituents: 2ml sample taken from the milk expressed as above (mixed sample)
**Shabnam et al 2021**[15]<br><br>**Parallel group RCT**	N = 70<br><br>University Hospital, Iran, 2016-2017	Infant: Born with BW between 2 and 2.5kg, without severe illness	Infants:<br>Mean PMA 36.5 – 37 weeks'<br><br>Mean BW 2.3kg	Intervention: 5-7 minute classical music<br><br>Instruction: Listen during feeds three times per day for four weeks. Both mother and infant should be able to listen to the music	Time spent breastfeeding: mother recorded daily for 28 days, advised to use timer<br><br>No other description given of how maternal reported outcomes were defined. All were recorded for 28 days<br><br>Weight/height and head circumference were measured by professionals on day 28
**Varisoglu et al 2020**[16]<br><br>**Parallel group RCT**	N = 44<br><br>University Hospital NICU, Turkey, 2017-2018	Primiparous<br><br>Infant: Born at 28 to 34 weeks' PMA and admitted to NICU	Mean age 28-29 years<br><br>Most were high school graduates<br><br>Infants:<br>Mean PMA 32 weeks'	Intervention: 15 minutes of calming Turkish music<br><br>Instruction: Listen twice a day while expressing for three consecutive days<br><br>Control: standard care	Milk quantity: volume expressed in 15 minutes during each of two daily sessions (morning and afternoon).<br><br>Spielberger State Trait Anxiety Index: self-report at baseline and on day four.<br><br>Maternal saliva cortisol: sample collected at baseline and on day four

© 2024 Levene I et al. *JAMA Pediatrics.*

Study ID and design	Setting	Key inclusion criteria	Key baseline characteristics	Intervention and control conditions	Outcomes included
**Yu et al 2019**[17]<br><br>**Crossover RCT**	N = 20<br><br>Urban China, 2018	Primiparous, 23-45 years, currently breastfeeding, non-smoker<br><br>Infant:<br>Less than two years old	Mean age 32 years<br><br>Infants:<br>Mean age 7.6 months	Intervention 1: lactation focused recording (including muscle relaxation and guided imagery), shortened from commercial recording and translated into Mandarin<br><br>Intervention 2: music (choice of new age or traditional Chinese)<br><br>Instruction: sit down in a specific comfortable room without the infant for 10 minutes, once only for each treatment (separated by 1-3 days)<br><br>Control: no stimuli. Note several other intervention arms not included in review	Visual analogue scale: 10cm visual scale anchored as completely relaxed to completely unrelaxed. Participant report before and immediately after the 10 minute treatment. Measured in mm and scored as 0-10<br><br>Physiological measurements of systolic blood pressure, diastolic blood pressure, heart rate and fingertip temperature, measured before and immediately after the 10 minute treatment.
**Yu et al 2023**[18,19]<br><br>**Parallel group RCT**	N = 96<br><br>Urban China, 2019-2020	Primiparous, intention to exclusively breastfeed for at least two months, non-smoker<br><br>Infant: singleton born at 34 to 37 weeks' PMA	Mean age 30 years<br><br>70% had a Bachelor's degree or higher<br><br>42% were in the lowest income bracket<br><br>37% caesarean birth<br><br>Infants: mean PMA 36.1 weeks'	Intervention: lactation focused recording (including muscle relaxation and guided imagery). Modified from a commercial recording and translated into Mandarin<br><br>Instruction: listen while breastfeeding or expressing milk at least daily for 7 weeks<br><br>Control: standard care.<br><br>Neither group was aware that they were in a randomised trial or that other participants were receiving/not receiving relaxation.	Outcomes measured at two and six weeks after birth<br><br>24 hour milk intake: test weighing infant before and after each feed for 48 hours (plus volume of breastmilk bottle feeds)<br><br>Infant diary: three-day diary record, recorded at fifteen minute epochs<br><br>Infant weight: measured on a digital scale. Standard deviation scores (SDS) using the Intergrowth-21 growth charts<br><br>Chinese version of Cohen's Perceived Stress Scale<br><br>Chinese version of Beck Anxiety Index<br><br>Breastmilk constituents: participants asked to express with a hand pump the first 20ml (foremilk)

BW = birthweight. NICU = neonatal intensive care unit. PMA = post-menstrual age. RCT = randomised controlled trial.

© 2024 Levene I et al. *JAMA Pediatrics.*

eTable 3: Summary of findings (high or moderate certainty outcomes)

Outcome	Number of participants (studies)	Example without relaxation (pooled from control groups)	Example with relaxation (95% CI)	Effect estimate (95% CI)	Certainty (downgrade reasons)	Comments
**Breastmilk and breastfeeding outcomes**						
**Milk protein**	205 (3 studies)	6 g/day† (2-3 months after late preterm/term birth)	No change	MD: 0.00 g/100 mL (0.00 to 0.00)	High	No change
**Milk carbo-hydrate**	139 (2 studies)	42 g/day† (2-3 months after late preterm/term birth)	0.9 g more (0.1 to 1.8g more)	MD: 0.15 g/100 mL (0.01 to 0.29)	Moderate (due to risk of bias)	Small increase of uncertain clinical significance
**Milk energy**	139 (2 studies)	400 kcal/day† (2-3 months after late preterm/term birth)	11.2 kcal more (0.6 to 21.8 kcal more)	MD: 1.84 kcal/100 mL (0.10 to 3.57)	Moderate (due to risk of bias)	Small increase of uncertain clinical significance
**Breastmilk quantity**	464 (10 studies)	611 g/day (late preterm/term baby, breastfeeding at 2-3 months old)	123 g higher (96 to 150g higher)	SMD: 0.73 (0.57 to 0.89)	Moderate (due to risk of bias)	Clinically significant, moderate increase
		396 mL/day (very preterm baby, expressed milk yield at 2-3 weeks after birth)	252 mL higher (197 to 307ml higher)			
**Mental health and physiology (parent)**						
**Physiology – example is diastolic blood pressure**	20 (1 study)	80 mmHg*	6 mmHg lower (3 to 9 mmHg lower)	MD: -5.9 mmHg (-9.1 to -2.8)	Moderate (due to indirectness)	Small decrease of uncertain clinical significance
**Stress**	355 (6 studies)	Perceived Stress Scale of 17 (2-3 months after late preterm/term birth)	Reduce to 14 (reduce to 13 to 16)	SMD: -0.49 (-0.70 to -0.27)	Moderate (due to imprecision)	Clinically significant, small decrease
**Anxiety**	399 (7 studies)	Beck Anxiety Index of 8 (2-3 months after late preterm/term birth)	Reduce to 5 (reduce to 3 to 6)	SMD: -0.47 (-0.66 to -0.29)	Moderate (due to risk of bias)	Small decrease of uncertain clinical significance
**Growth and behaviour outcomes (infant)**						
**Weight**	226 (3 studies)	Infant on 50th centile*	Increase to 69th centile (increase to 62nd to 76th centile)	MD: 0.51 SDS change (0.30 to 0.72)	Moderate (due to imprecision)	Clinically significant, moderate increase
		Infant on 5th centile	Increase to 13th centile (increase to 9th to 18th centile)			

© 2024 Levene I et al. *JAMA Pediatrics.*

Outcome	Number of participants (studies)	Example without relaxation (pooled from control groups)	Example with relaxation (95% CI)	Effect estimate (95% CI)	Certainty (downgrade reasons)	Comments
**Length**	226<br>(3 studies)	Infant on 50th centile*	Increase to 52nd centile (reduce to 42nd to increase to 61st centile)	MD: 0.04 SDS change (-0.21 to 0.29)	Moderate<br>(due to inconsistency)	No change
		Infant on 5th centile	Increase to 6th centile (reduce to 3rd to increase to 9th centile)			

SMD = standardised mean difference; RR = risk ratio. MD = mean difference. †combining pooled control milk intake of 611g per day with pooled control milk energy of 65 kcal/100ml, protein of 0.9g/100mL or carbohydrate of 6.8g/100mL. 1ml and 1g of human milk are near equivalent.
*Population averages.

© 2024 Levene I et al. *JAMA Pediatrics.*

eTable 4: Summary of findings (low or very low certainty)

Outcome	Number of participants (studies)	Effect estimate (95% CI)	Certainty (downgrade reasons)	Comments: clinical significance
**Breastmilk and breastfeeding outcomes**				
Exclusive breastmilk	651 (2 studies)	RR: 0.99 (0.90 to 1.09)	Low (due to risk of bias, imprecision)	No change
Any breastmilk	47 (1 study)	RR: 1.18 (0.89 to 1.58)	Low (due to risk of bias, imprecision)	No change
Milk fat	387 (5 studies)	SMD: 0.17 (-0.04 to 0.37)	Low (due to risk of bias, imprecision)	No change
Milk cortisol	63 (1 study)	MD: -44.5 % (-76.1 to -12.9)	Low (due to risk of bias, imprecision)	Moderate decrease of uncertain clinical significance
Expressing frequency	51 (1 study)	MD: 1 per day (-1 to 1.5)	Low (due to risk of bias, imprecision)	No change
Breastfeeding self-efficacy	60 (1 study)	Median Difference: 0* (-7.5 to 5)	Low (due to risk of bias, imprecision)	No change
Time spent breastfeeding	58 (1 study)	MD: 124.9 minutes (103.3 to 146.6)	Very low (2 levels: risk of bias; 1 level imprecision)	Large increase of uncertain clinical significance
Breastfeeding frequency	18 (1 study)	MD: 3.9 per day (1.1 to 6.7)	Very low (2 levels: risk of bias; 1 level imprecision)	Small increase of possible clinical significance
Time to lactogenesis II	62 (1 study)	MD: -1.1 days (-1.7 to -0.6)	Very low (2 levels: risk of bias; 1 level imprecision)	Moderate decrease of possible clinical significance
Colostrum score	304 (1 study)	RR 1.6 (1.2 to 2.0)	Very low (2 levels: risk of bias; 1 level imprecision; indirectness)	Small increase of minimal clinical significance
**Growth and behaviour outcomes (infant)**				
Time sleeping	179 (4 studies)	MD: 53.6 minutes (41.8 to 65.3)	Low (2 levels: risk of bias)	Clinically significant, moderate increase
Time crying/fussing	108 (3 studies)	MD: -5.2 minutes (-30.8 to 20.4)	Low (2 levels: risk of bias)	No change
Time awake and alert	146 (3 studies)	MD: 38.3 minutes (18.2 to 58.4)	Very low (2 levels: risk of bias; 1 level inconsistency)	Small increase of possible clinical significance
Head circumference	58 (1 study)	MD: 0.27 cm (-0.53 to 1.07)	Very low (2 levels: risk of bias; 1 level imprecision)	No change
Fat free mass	29 (1 study)	MD: 0.5 kg (-0.06 to 1.06)	Very low (2 levels: risk of bias; 1 level imprecision)	No change
**Mental health and stress related outcomes (mother/lactating parent)**				
Perceived relaxation	20 (1 study)	MD: 1.1 (0.43 to 1.7)	Low (due to risk of bias, imprecision)	Small increase of possible clinical significance
Depression	143 (3 studies)	SMD: -0.23 (-0.56 to 0.10)	Low (due to risk of bias, imprecision)	No change
Salivary cortisol	168 (4 studies)	MD: -0.34 nmol/L (-0.73 to 0.05)	Very low (2 levels: risk of bias; 1 level imprecision)	No change

SMD = standardised mean difference; RR = risk ratio. MD = mean difference. *Breastfeeding self-efficacy Scale (short form for NICU)

© 2024 Levene I et al. *JAMA Pediatrics.*

eTable 5: Data for individual meta-analysis outcomes

	Outcome	N	Mean difference	95% confidence interval	Risk of Bias
**Human milk quantity**					
**Ak 2015**[1]	15 minutes of expression on day 4 of study (day after birth not reported)	29	0.5 mL	0.2 to 0.8*	High
**Dabas et al 2019**[3]	One expressing session on mean day 14 after birth (averaged from 24hr record)	50	15.1 mL	3.5 to 26.7	Some concerns
**Dib et al 2022**[4]	24 hour intake from the breast at 6-8 weeks after birth	7	184 g	-162 to 530	High
**Feher et al 1989**[6]	One expressing session (no specified length) on approximately day 11 after birth	55	34.7 mL	6.1 to 63.3	High
**Keith et al 2012**[7]	24 hour expressed milk record on day 14 of study (approximately day 16 after birth)	120	396.1 mL[†]	276.9 to 515.2	Some concerns
**Massa et al 2022**[9]	24 hour expressed milk record on day 9 after birth	54	132.2 mL	-99.3 to 363.7	Some concerns
**Mohd Shukri et al 2019**[10]	24 hour intake from the breast (averaged over two weeks) at 12-14 weeks after birth	19	145.0 g	-60.1 to 350.1	Some concerns
	24 hour intake from the breast (averaged over two weeks) at 12-14 weeks after birth, adjusted for infant sex and intake at 2 weeks after birth	19	226.5 g	24.0 to 430.0	Some concerns
**Sefid Haji et al 2022**[14]	15 minutes of expression on day 6 after birth	66	4.9 mL	2.5 to 7.3	Low
	Change between 15 minutes of expression on day 1 and 6 after birth	66	5.0 mL	4.2 to 5.7	Low
**Varisoglu et al 2020**[16]	15 minutes of expression on day 4 of study (day after birth not reported)	38	16.2 mL**	-0.3 to 32.7	High
	Change between 15 minutes of expression on day 3 and day 4 of study (day after birth not reported)	38	10.8 mL	2.5 to 19.1	High
**Yu et al 2023**[18]	24 hour intake from the breast (averaged over 48 hours) at 7-9 weeks after birth	27	1.0 g	-28.5 to 30.5	High
**Human milk fat**					
**Dib et al 2022**[4]	Change in composite milk fat from week 2-3 to week 6-8 after birth at around 11am (g/100 mL)	47	0.35	-0.6 to 1.2	Some concerns
	Composite milk fat at week 6-8 after birth at around 11am (g/100 mL)	56	-0.1	-0.8 to 0.5	Some concerns
**Feher et al 1989**[6]	Composite milk creamatocrit on approximately day 11 after birth, unspecified circadian timing (%)	45	0.40	-1.0 to 1.8	High
**Keith et al 2012**[7]	Composite milk fat on day 14 of study at around midday (day 16 after birth; g/L)	119	10.7[†]	-32.4 to 53.8	Some concerns
**Sefid Haji et al 2022**[14]	Change in composite milk triglyceride from day 1 to 6 after birth at around midday (mg/dL)	66	32.9	25.5 to 40.3	Low
	Composite milk triglyceride on day 6 after birth at around midday (mg/dL)	66	28.8	-9.3 to 66.8	Low
**Yu et al 2023**[18]	Change in foremilk fat from week 1 to 8 after birth at around 10am (g/100 mL)	92	0.12	-0.0 to 0.3	Low
	Foremilk fat at week 8 after birth at around 10am (g/100 mL)	92	0.12	-0.1 to 0.3	Low

© 2024 Levene I et al. *JAMA Pediatrics.*

	Outcome	N	Mean difference	95% confidence interval	Risk of Bias
**Infant weight, length and head circumference**					
**Dib et al 2022**[4]	Change in weight SDS from 2 to 6-8 weeks after birth	67	0.40	0.09 to 0.71	Low
	Change in length SDS from 2 to 6-8 weeks after birth	55	-0.2	-0.65 to 0.25	Some concerns
**Mohd Shukri et al 2019**[10]	Change in weight SDS from 2 to 12 weeks after birth	63	0.76	0.29 to 1.23	Low
	Change in length SDS from 2 to 12 weeks after birth	63	-0.09	-0.52 to 0.34	Low
**Shabnam et al 2021**[15]	Weight at 28 days (grams)	58	492.4	303.2 to 681.6	High
	Length at 28 days (cm)	58	4.89	3.41 to 6.37	High
	Head circumference at 28 days (cm)	58	0.27	-0.53 to 1.07	High
**Yu et al 2023**[18]	Change in weight SDS from 1 to 8 weeks after birth	96	0.51	0.15 to 0.87	Low
	Change in length SDS from 1 to 8 weeks after birth	96	0.38	-0.1 to 0.8	Low
**Infant time awake and alert (not crying/fussing)**					
**Mohd Shukri et al 2019**[10]	Time (mins) per day (averaged from three day diary of 15 minute epochs) at 6-8 weeks after birth	37	-56.0	-131.0 to 19.0	High
**Shabnam et al 2021**[15]	Time (hours per day) averaged over 28 days	58	0.8	0.5 to 1.1	High
**Yu et al 2023**[18]	Time (mins) per day (averaged from three day diary of 15 minute epochs) at 8 weeks after birth	51	19.4	-48.5 to 87.2	High
**Infant time sleeping**					
**Dib et al 2022**[4]	Time (mins) per day (averaged from three day diary of 15 minute epochs) at 6-8 weeks after birth	33	-4.4**	-102.5 to 93.7	High
**Mohd Shukri et al 2019**[10]	Time (mins) per day (averaged from three day diary of 15 minute epochs) at 6-8 weeks after birth	37	82.0	16.0 to 149.0	High
**Shabnam et al 2021**[15]	Time (hours) "during 28 days" (unclear format or period of recording)	58	0.9	0.69 to 1.12	High
**Yu et al 2023**[18]	Time (mins) per day (averaged from three day diary of 15 minute epochs) at 8 weeks after birth	51	32.7	-49.2 to 114.5	High
**Infant time crying/fussing**					
**Dib et al 2022**[4]	Time (mins) per day (averaged from three day diary of 15 minute epochs) at 6-8 weeks after birth Time (mins)	31	-22.6**	-75.1 to 29.9	High
**Mohd Shukri et al 2019**[10]	Time (mins) per day (averaged from three day diary of 15 minute epochs) at 6-8 weeks after birth	37	21.0	-27.0 to 69.0	High
**Yu et al 2023**[18]	Time (mins) per day (averaged from three day diary) at 8 weeks after birth	41	-14.0	-53.2 to 24.3	High
**Anxiety (lactating mother/parent)**					
**Dabas et al 2019**[3]	Perinatal Anxiety Screening Scale on mean day 14 after birth	50	-8.4	-13.7 to -3.1	Some concerns
**Massa et al 2022**[9]	Spielberger State Trait Anxiety Index on day 10-16 after birth	60	1.8	-9.8 to 13.4	Some concerns
**Mohd Shukri et al 2019**[10]	Beck Anxiety Index at 12-14 weeks after birth	61	-3.0	-8.6 to 2.6	Some concerns
**Perez-Blasco et al 2013**[12]	Depression, Anxiety and Stress (anxiety subscale) at mean 13 months after birth	21	-4.8	-8.3 to -1.2	Some concerns
**Varisoglu et al 2020**[16]	Spielberger State Trait Anxiety Index on day 4 of the study (day after birth not reported)	38	-6.1	-9.3 to -2.9	High

© 2024 Levene I et al. *JAMA Pediatrics.*

	Outcome	N	Mean difference	95% confidence interval	Risk of Bias
**Yu et al 2023**[18]	Beck Anxiety Index at 8 weeks after birth	96	-1.4	-3.4 to 0.6	Some concerns
	Change in Beck Anxiety Index between week 1 and week 8 after birth	96	-1.3	-3.0 to 0.4	Some concerns
**Stress (lactating mother/parent)**					
**Dabas et al 2019**[3]	Perceived Stress Scale: NICU on mean day 14 after birth	50	-0.7	-1.0 to -0.4	Some concerns
**Dib et al 2022**[4]	Perceived Stress Scale at 6-8 weeks after birth	67	-1.7	-4.3 to 0.9	Some concerns
	Change in Perceived Stress Scale from 2 to 6-8 weeks after birth	67	-0.2	-2.8 to 2.4	Some concerns
**Massa et al 2022**[9]	Perceived Stress Scale: NICU on day 10-16 after birth	60	1.9	-10.3 to 14.1	Some concerns
**Mohd Shukri et al 2019**[10]	Perceived Stress Scale at 12-14 weeks after birth	61	-3.1	-5.9 to -0.3	Some concerns
**Perez-Blasco et al 2013**[12]	Depression, Anxiety and Stress (stress subscale) at mean 13 months after birth	21	-8.5	-15.1 to -1.8	Some concerns
**Yu et al 2023**[18]	Perceived Stress Scale at 8 weeks after birth	96	-2.8	-5.3 to -0.2	Some concerns
	Change in Perceived Stress Scale from 1 to 8 weeks after birth	96	-2.7	-4.5 to -0.8	Some concerns
**Depression (lactating mother/parent)**					
**Dib et al 2022**[4]	Edinburgh Postnatal Depression Scale at 6-8 weeks after birth	61	-0.3	-2.4 to 1.8	Some concerns
	Change in Edinburgh Postnatal Depression Scale from 2 to 6-8 weeks after birth	54	0.5	-1.3 to 2.3	Some concerns
**Massa et al 2022**[9]	Edinburgh Postnatal Depression Scale on day 10-16 after birth	60	Median difference: -1	-5 to 2.5	Some concerns
**Mohd Shukri et al 2019**[10]	Edinburgh Postnatal Depression Scale at 12-14 weeks after birth	61	-1.3	-3.3 to 0.7	Some concerns
**Perez-Blasco et al 2013**[12]	Depression, Anxiety and Stress (depression subscale) at mean 13 months after birth	21	-1.2	-4.3 to 1.9	Some concerns
**Salivary cortisol (lactating parent/mother)**					
**Ak 2015**[1]	Units: nmol/L.<br>Collected on day 4 of the study (day after birth not reported)	29	0.08	-0.44 to 0.59[¥]	High
**Dib et al 2022**[4]	Units: microgram/dL.<br>Collected at 6-8 weeks	49	0.02	-0.02 to 0.06	High
	Units: microgram/dL.<br>Change from 2 to 6-8 weeks	41	-0.08	-0.15 to -0.01	High
**Mohd Shukri et al 2019**[10]	Units: microgram/dL.<br>Collected at 6 weeks after birth	60	-0.27	-0.78 to 0.24	Some concerns
**Varisoglu et al 2020**[16]	Units: nmol/l.<br>Collected on day 4 of the study (day after birth not reported)	38	-0.44	-1.08 to 0.21	High

Composite milk is a mixture of foremilk and hindmilk. *Confidence interval calculated by review authors using paired test p value reported in publication (crossover trial) †pooled intervention groups (identified B and D in original report) **provided by authors on request ¥Assuming unpaired data – conservative approach

eFigure 1: PRISMA Flowchart for systematic search update

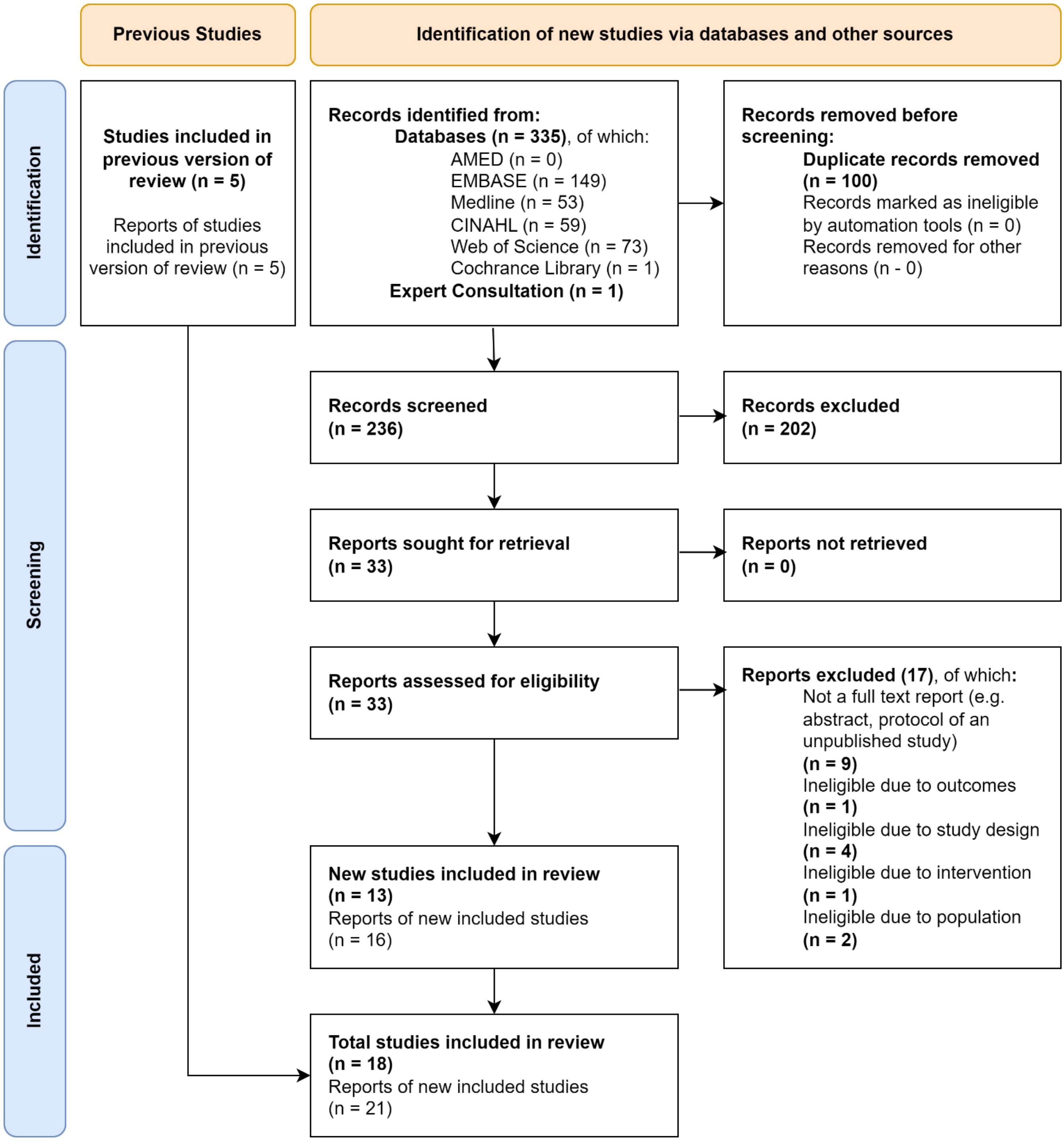

© 2024 Levene I et al. *JAMA Pediatrics.*

eFigure 2: Duration of relaxation intervention across studies

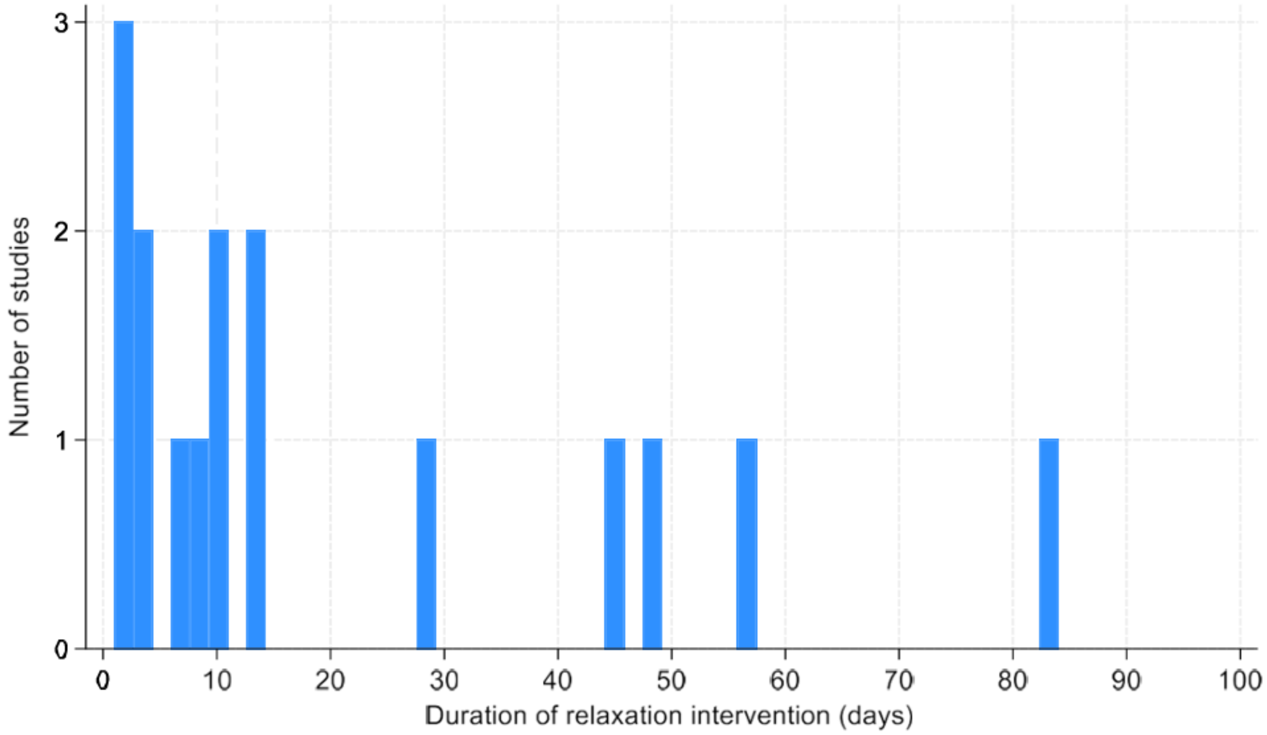

eFigure 3: Funnel plots for individual meta-analyses with more than four studies included

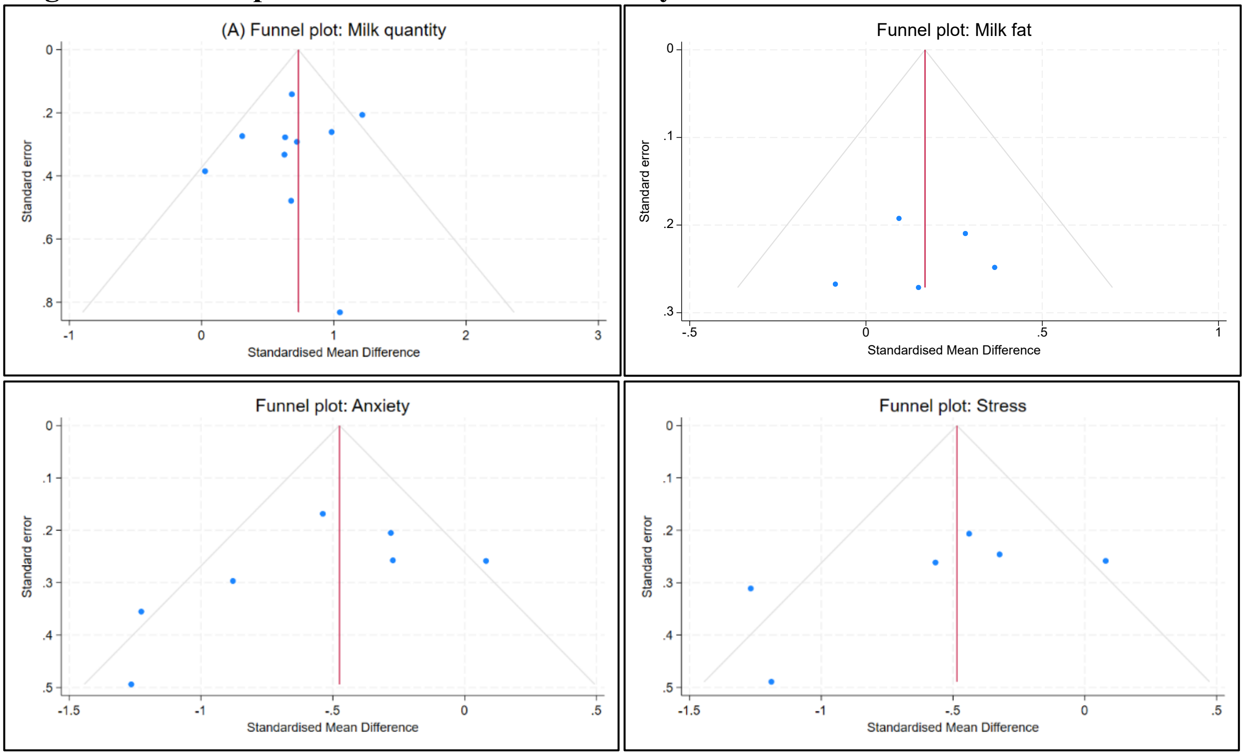

© 2024 Levene I et al. *JAMA Pediatrics.*

eFigure 4: Forest plots for outcomes with low and very low certainty evidence, grouped by risk of bias assessment

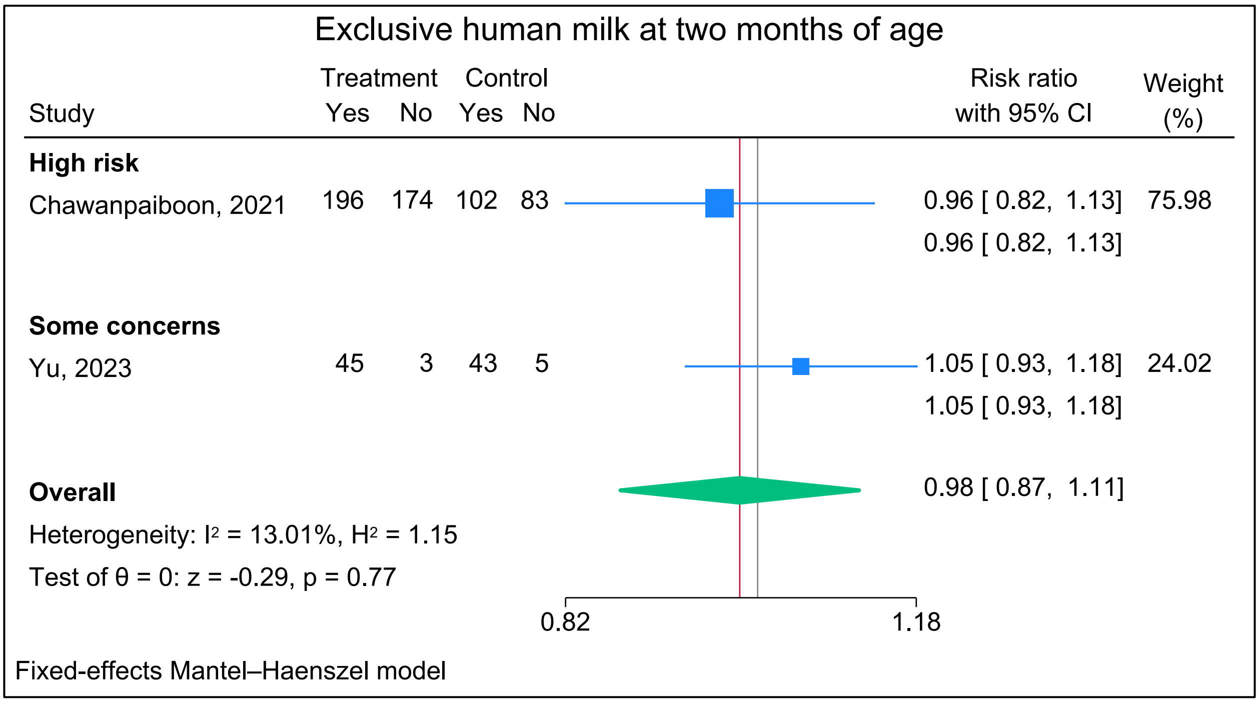

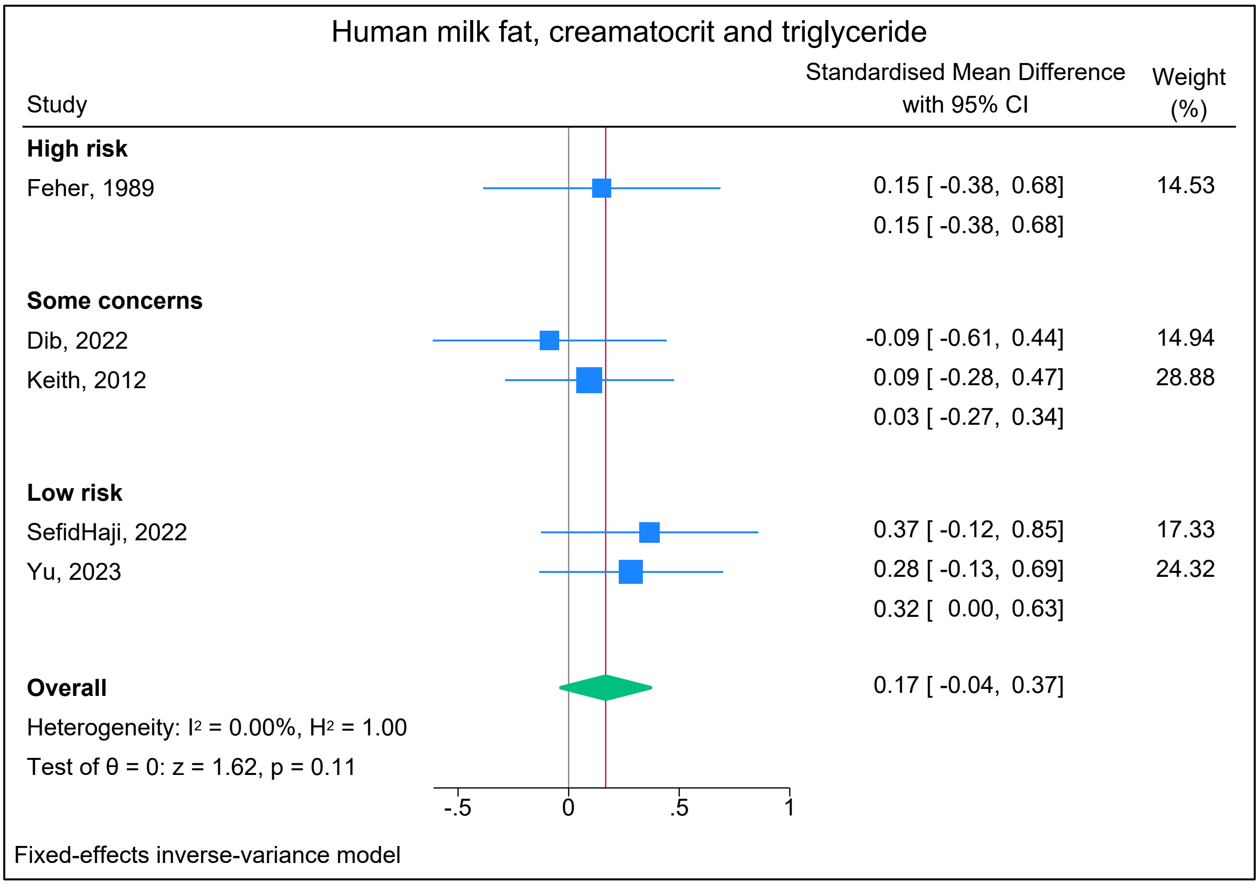

© 2024 Levene I et al. *JAMA Pediatrics.*

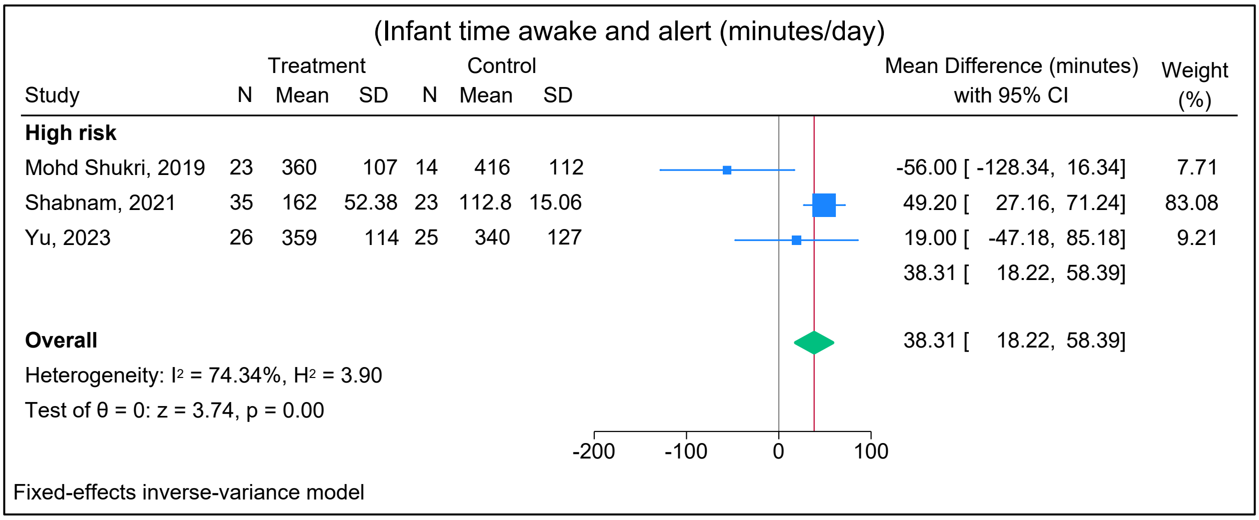

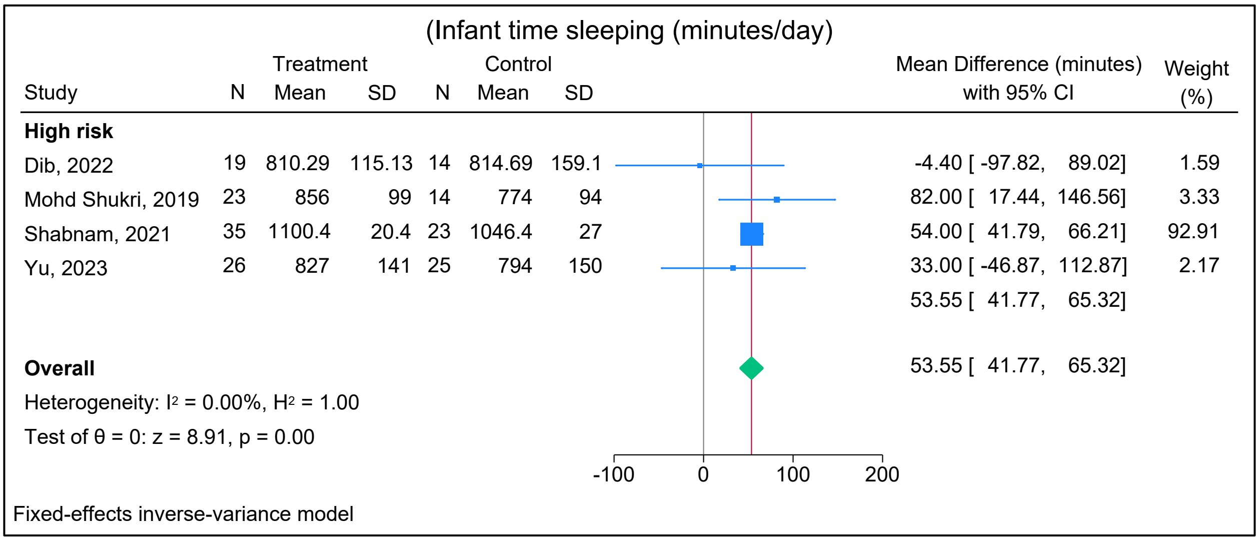

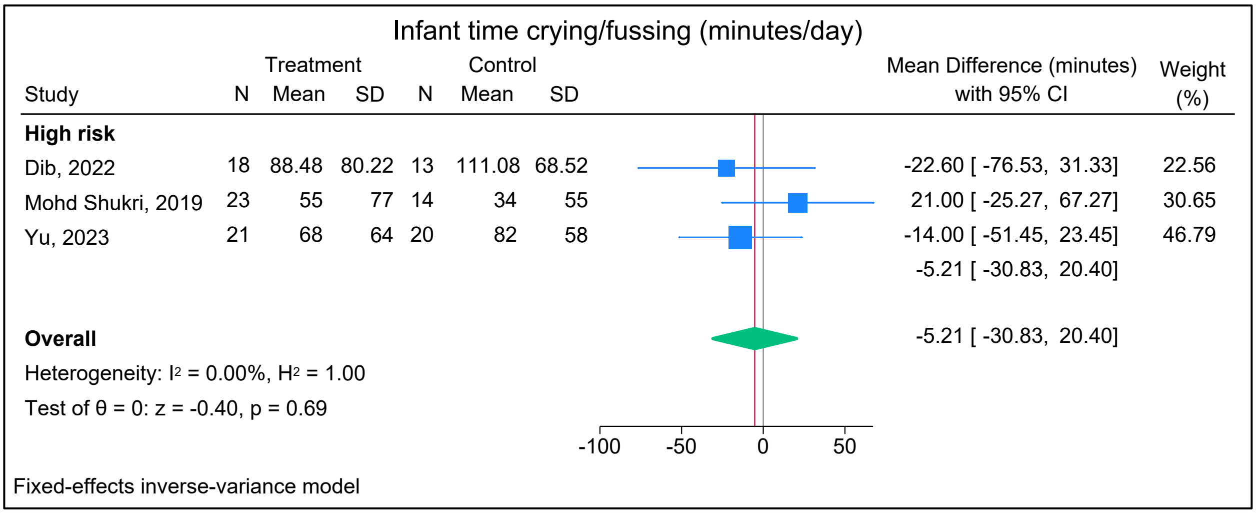

© 2024 Levene I et al. *JAMA Pediatrics.*

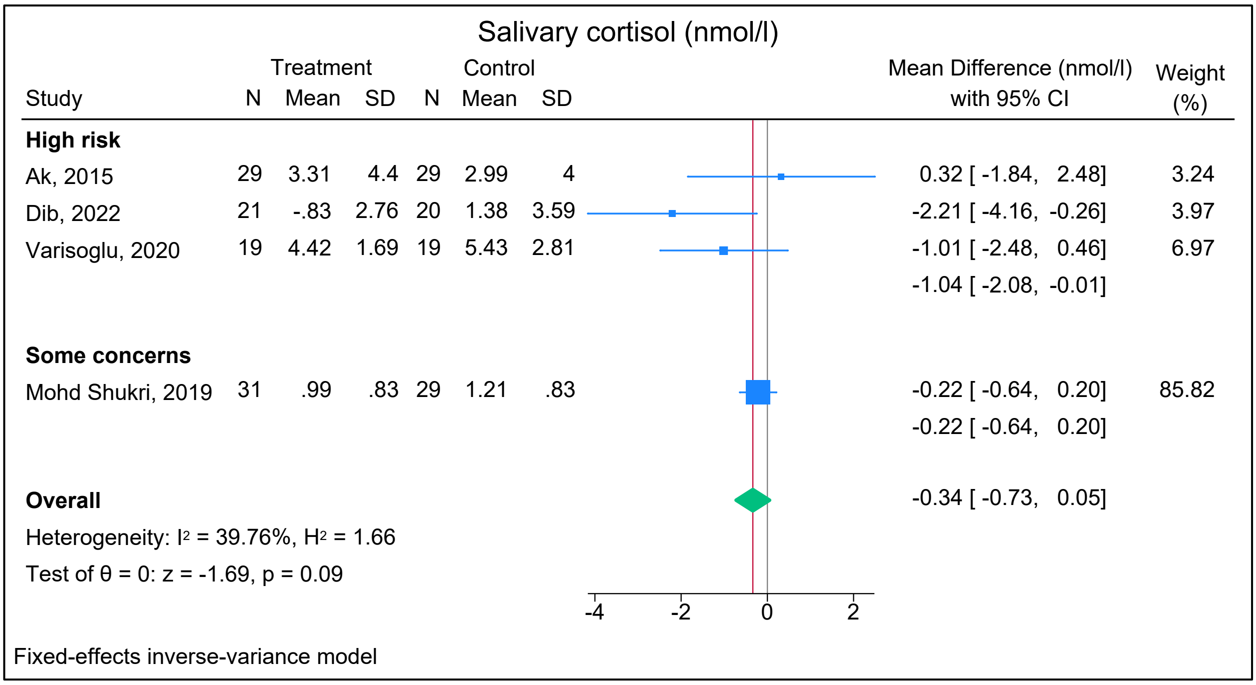

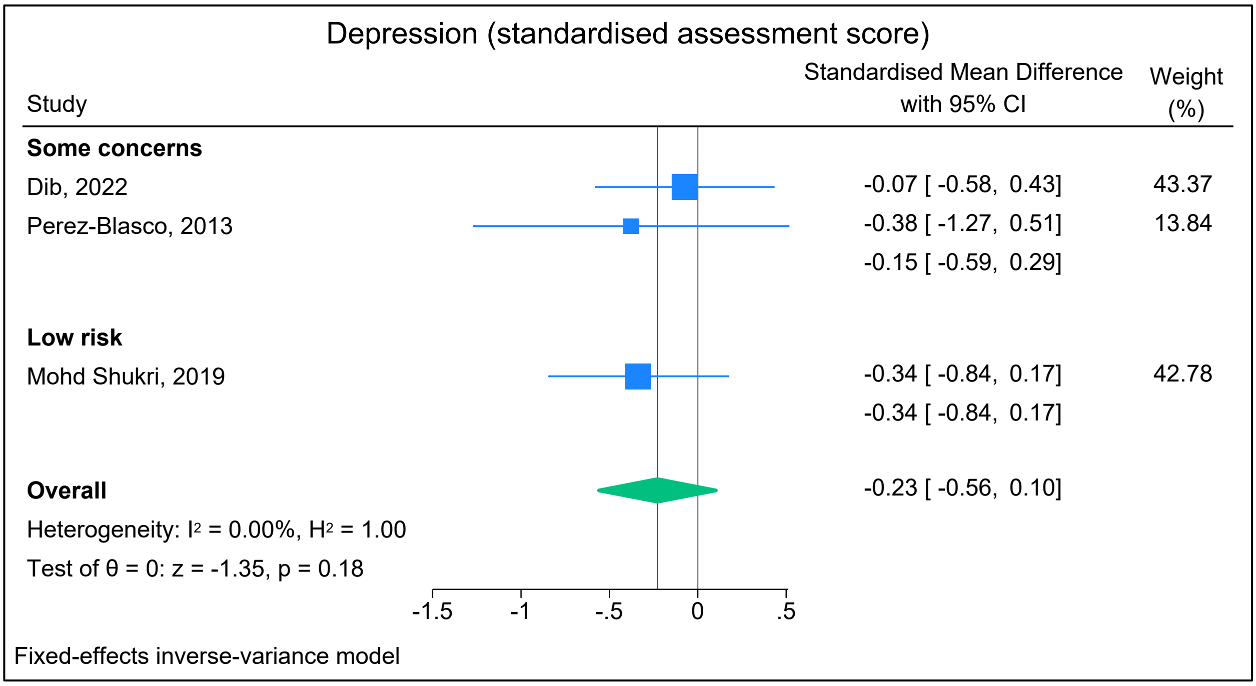

© 2024 Levene I et al. *JAMA Pediatrics.*

eFigure 5: Forest plots for sub-group analysis of human milk quantity

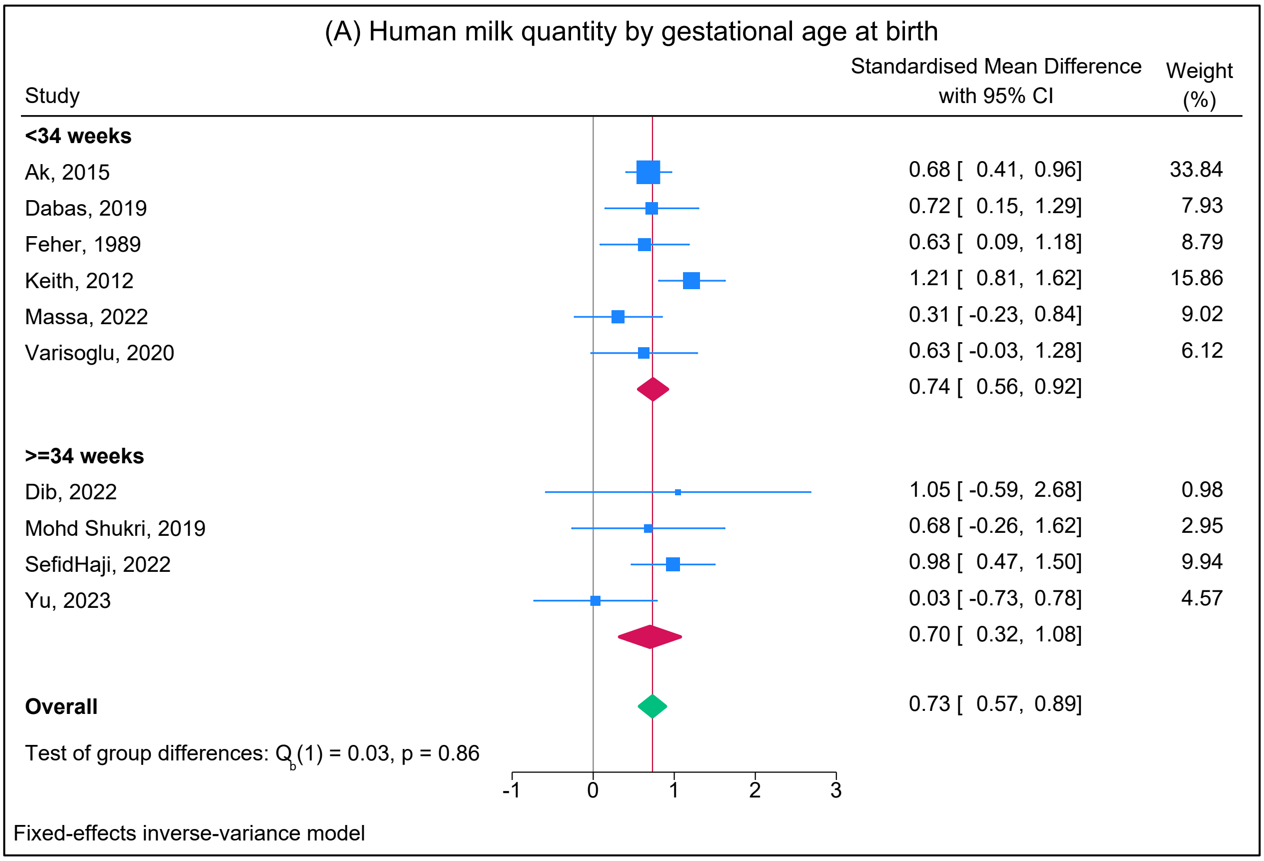

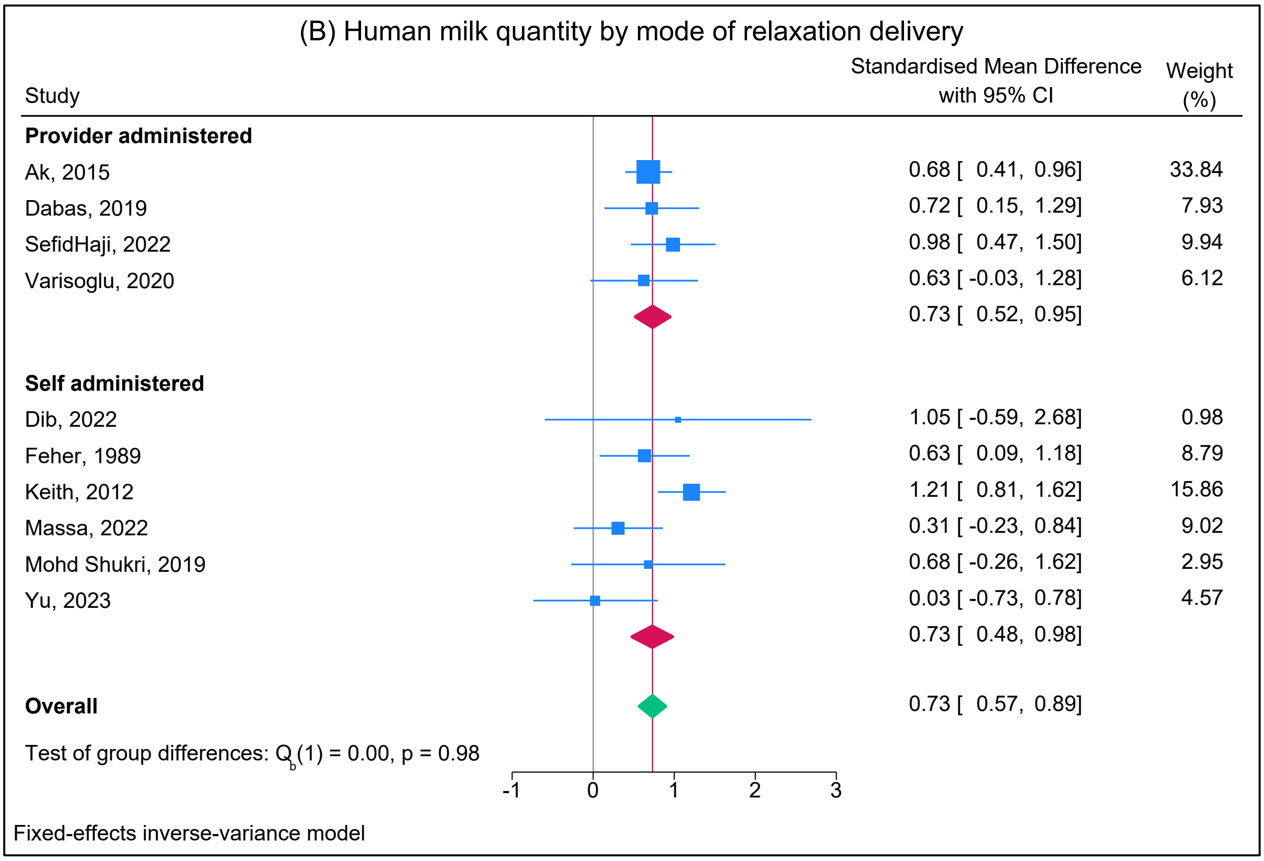

© 2024 Levene I et al. *JAMA Pediatrics.*

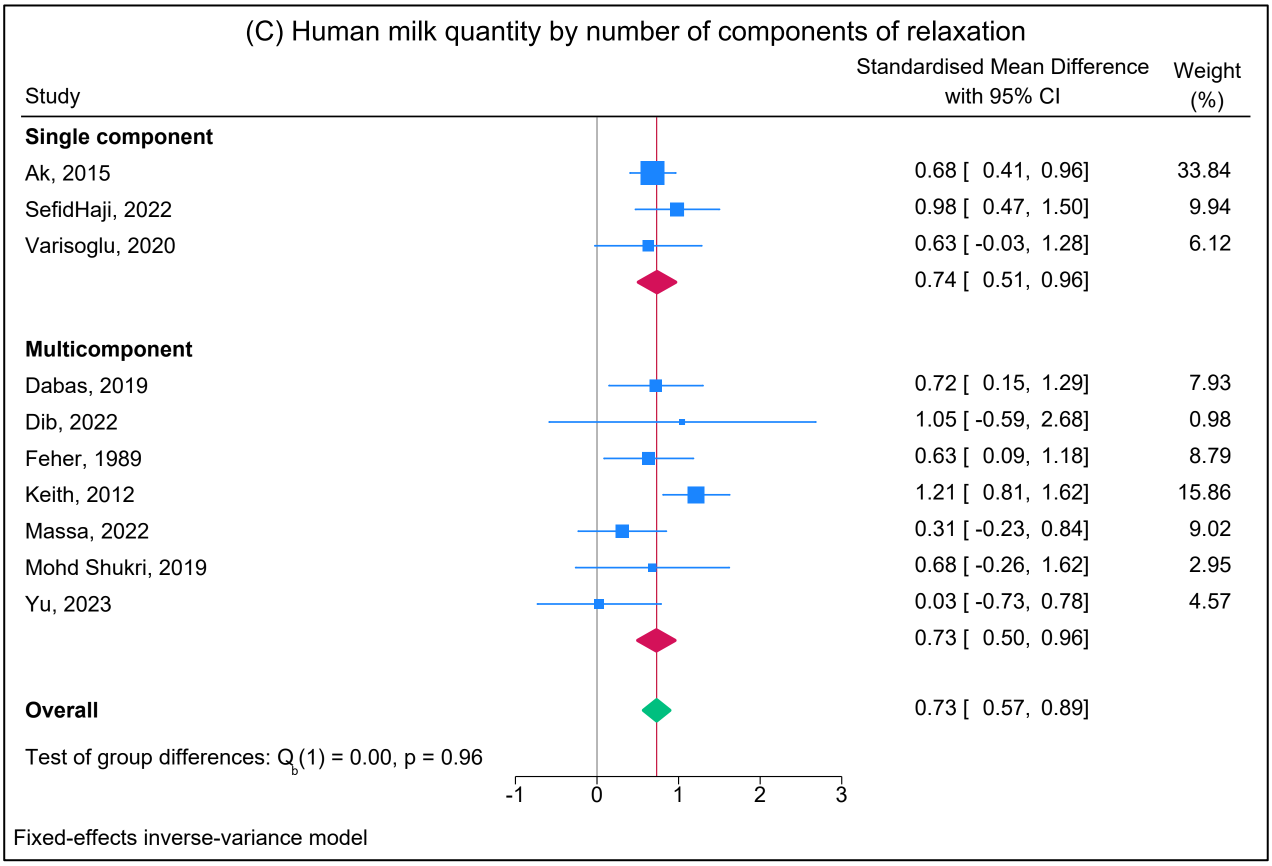

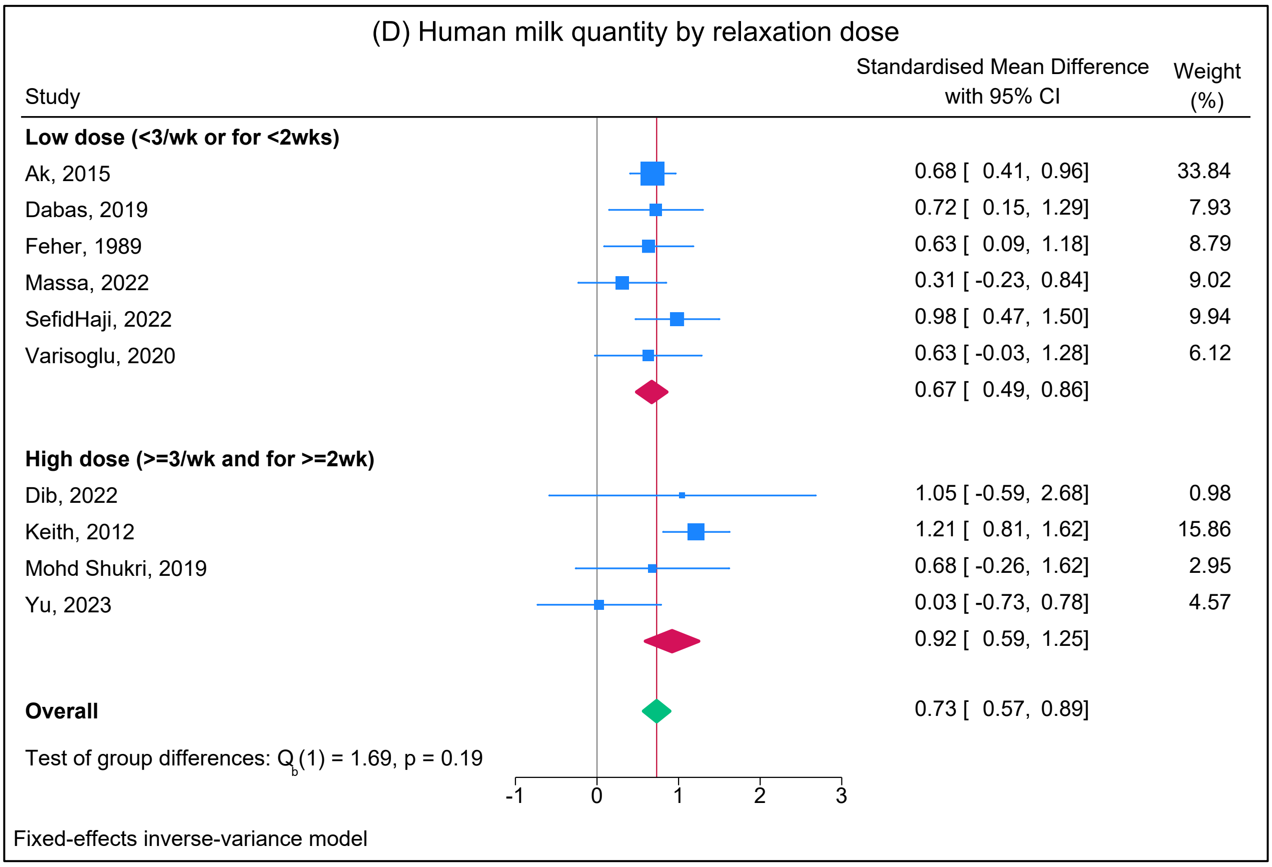

© 2024 Levene I et al. *JAMA Pediatrics.*

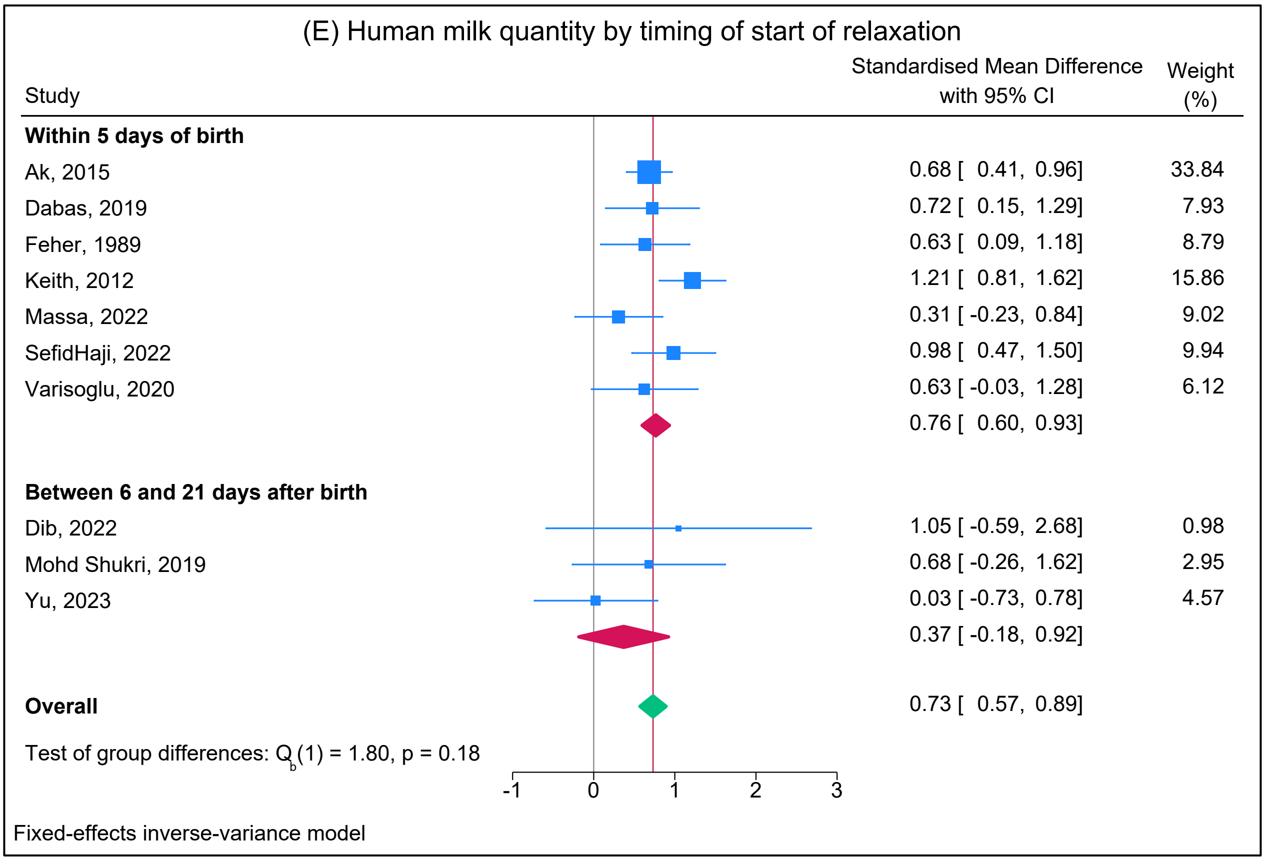

© 2024 Levene I et al. *JAMA Pediatrics.*

eAppendix: Evidence summary for outcomes with low/very low certainty not reported in the main text

Primary Outcomes

There was low certainty evidence of no difference in **milk fat** (SMD 0.17, 95% CI -0.04 to 0.37; Five studies, 388 participants). Five RCTs contributed to this outcome, reporting data on milk fat[4,18], creamatocrit[6,7] and triglyceride[14] after between six days and eight weeks of music[14] or lactation-specific guided relaxation[4,6,7,18]. Composite (mixed)[4,6,7,14] or foremilk[18] samples were taken between 10am and noon[4,7,14,18] or at an unspecified time[6]. Three studies took place in the NICU setting[6,7,14] and two in the community, after late preterm or early term birth[4,18]. One study was at high risk of bias due to concern over allocation concealment and unequal missing data[6]. Two others had some concern over risk of bias[4,7]. GRADE assessment: low certainty (downgraded by one level for risk of bias and one level for imprecision).

There was low certainty evidence of a reduction in **hindmilk cortisol** after the first exposure to relaxation (-44.5%, 95% CI -76.1 to -12.9; 63 participants), but not after six weeks of regular use, in a single study. This study was classified as some concern for risk of bias due to a change in the format of the outcome compared to the protocol[10]. GRADE assessment: low certainty (downgraded by one level for risk of bias and one level for imprecision).

There was very low certainty evidence for no change in **infant head circumference** in a single study at high risk of bias[15] (MD 0.27cm, 95% CI -0.53 to 1.07, 58 participants). GRADE assessment: very low certainty (downgraded by one level for imprecision and two levels for risk of bias).

There was low certainty evidence for no change in **infant body composition** in a single study at high risk of bias due to large and unbalanced loss to follow up[10] (MD for fat free mass of 0.5kg, 95% CI -0.06 to 1.06; 29 participants). GRADE assessment: very low certainty (downgraded by two levels for risk of bias and one level for imprecision).

Four RCTs[4,10,15,18] reported infant behaviour at between four and eight weeks of age, although not all studies reported on all categorisations of behaviour. All studies were at high risk of bias due predominantly to large and unbalanced loss to follow up. However, one study[15] was particularly high risk, with significant concerns in four out of five domains.

There was very low certainty for an increase in **infant time awake and alert** (MD 38.3 minutes, 95% CI 18.2 to 58.4, $p<0.001$; three studies, 146 participants). There was substantial heterogeneity in this analysis, with I^2 of 74%, and the summary estimate was dominated by the study with the highest number of domains assessed as high risk of bias[15]. If this study were excluded, there would be no evidence for a difference in time spent awake and alert (MD -15.2 minutes, 95% CI -64 to 33.7, 88 participants). In addition, sensitivity analysis using random effects meta-analysis gave a different conclusion from fixed effects analysis, showing no evidence of a change in time spent awake (MD 11.4 minutes, 95% CI -48.8 to 71.5). Both alternative analyses casts significant doubt on a conclusion that relaxation increases infant

© 2024 Levene I et al. *JAMA Pediatrics.*

time spent awake. GRADE assessment: very low certainty (downgraded by two levels for risk of bias and one level for inconsistency).

There was low certainty evidence for a moderate increase in **infant sleep** (MD 53.6 minutes, 95% CI 41.8 to 65.3, p<0.001; four studies, 179 participants). When the dominant study with highest risk of bias[15] was excluded, the effect estimate remained of similar size (MD of 47.6 minutes, 95% CI 3.4 to 91.8, p<0.001, 121 participants). GRADE assessment: low certainty (downgraded by two levels for risk of bias).

There was low certainty evidence for no change in **infant crying time** (MD of -5.2 minutes, 95% CI -30.8 to 20.4; three studies, 108 participants). GRADE assessment: low certainty (downgraded by two levels for risk of bias).

Secondary Outcomes

There was low certainty evidence for an increase in maternal **perceived relaxation** in a single study (MD visual assessment scale; VAS of 1.1, 95% CI 0.4 to 1.7, p<0.01, 20 participants). This was a crossover RCT testing a single exposure to relaxation[17]. The VAS scale was anchored at 1 to 10. This study had some concerns over risk of bias due to the inherent subjectivity of relaxation self-assessment in an unblinded study. GRADE assessment: low certainty (downgraded by one level for risk of bias and one level for imprecision).

There was very low certainty evidence for no change in maternal **salivary cortisol** after an episode of relaxation (MD -0.34, 95% CI -0.73 to 0.05; 4 studies, 168 participants). Four studies contributed to this outcome[1,4,10,16], two in a NICU setting with music[1,16] and two in the community with lactation-specific guided relaxation[4,10]. This outcome was classified as high risk of bias in three studies, due to inadequate allocation concealment and results selection[16], inadequate washout in a crossover study[1] or the high level of missing data[4]. One study was classified as some concerns over risk of bias due to differences in the format of outcome reporting compared to the protocol[10]. GRADE assessment: very low certainty (downgraded by two levels for risk of bias and one level for imprecision).

There was low certainty evidence for no change in maternal **depression** (SMD -0.23, 95% CI -0.56 to 0.10; 3 studies, 143 participants). Three studies contributed to this outcome[4,10,12], using two different standardised scales. This outcome was classified as some concerns for risk of bias in two studies[4,12] due to the inherent subjectivity of self-reported depression in an unblinded study, and due to missing data. One further study could not be included due to the format of data reported but also showed no difference in this outcome[9]. GRADE assessment: low certainty (downgraded by one level for risk of bias and one level for imprecision).

There was low certainty evidence for no difference in **breastfeeding self-efficacy** in a single study[9], using the Breastfeeding Self-Efficacy Scale short form for NICU after a week of recommended daily use of a mindfulness app (median difference 0, 95% CI -7.5 to 5, 1 study, 60 participants). This study is classified at high risk of bias due to missing data and the subjective nature of the measurement. GRADE assessment: low certainty (downgraded by two levels for risk of bias and imprecision).

© 2024 Levene I et al. *JAMA Pediatrics.*

There was very low certainty evidence for an increase in **breastfeeding frequency** in a single study[4] after provision of a guided relaxation and visualisation recording to mothers of late preterm and early term infants for about four weeks (MD 3.9 per day, 95% CI 1.13 to 6.67, 1 study, 18 participants). This outcome was at high risk of bias due to the level of missing data. GRADE assessment: very low certainty (downgraded by one level for risk of bias and one level for imprecision).

There was low certainty evidence for no change in **expressing frequency** in a single study[9], measured on day nine after birth after provision of a mindfulness app in the NICU setting (median difference 1, 95% CI -1 to 1.5, 1 study, 54 participants). This outcome was classified as some concerns for risk of bias due to missing data. GRADE assessment: low certainty (downgraded by one level for risk of bias and one level for imprecision).

There was very low certainty evidence for an increase in **time spent breastfeeding** in a single study[15] after use of instrumental music for low birth weight infants for four weeks (MD 124.9 minutes over 28 days, 95% CI 103.3 to 146.6, 1 study, 58 participants). This outcome was classified as high risk of bias due to missing data and unsatisfactory method of measurement. GRADE assessment: very low certainty (downgraded by two levels for risk of bias and one level for imprecision).

There was very low certainty for a reduction in the **time to secretory activation** ("lactogenesis II" or mature milk "coming in") in a single study[13] (MD -1.1 days, 95% CI -1.68 to -0.60, 1 study, 62 participants). This study used relaxing flute music for 30 minutes twice a day. This study was classified as high risk of bias due to possible reporting selection and missing data. GRADE assessment: very low certainty (downgraded by two levels for risk of bias and one level for imprecision).

There was very low certainty for an increase in early **colostrum quantity** in a single study[8] using a calming song in the delivery room (RR of the highest 'colostrum score' of 1.58, 95% CI 1.24 to 2.00, 1 study, 304 participants). The highest score meant that three or more drops of colostrum were obtained after a single compression after the first breastfeed and within 2 hours of birth. There were some concerns over risk of bias due to concerns with unequal measurement timing between allocation arms. GRADE assessment: very low certainty (downgraded by one level for each of risk of bias, imprecision and indirectness).

© 2024 Levene I et al. *JAMA Pediatrics.*

eReferences:

1. AK J. Impact of Music Therapy on Breast Milk Secretion in Mothers of Premature Newborns. *Journal of Clinical and Diagnostic Research*. 2015;9(4):CC04-CC06. doi:10.7860/JCDR/2015/11642.5776

2. Chawanpaiboon S, Titapant V, Pooliam J. A Randomized Controlled Trial of the Effect of Music During Cesarean Sections and the Early Postpartum Period on Breastfeeding Rates. *Breastfeeding Medicine*. 2021;16(3):200-214. doi:10.1089/bfm.2020.0299

3. Dabas S, Joshi P, Agarwal R, Yadav RK, Kachhawa G. Impact of audio assisted relaxation technique on stress, anxiety and milk output among postpartum mothers of hospitalized neonates: A randomized controlled trial. *Journal of Neonatal Nursing*. 2019;25(4):200-204. doi:10.1016/j.jnn.2019.03.004

4. Dib S, Wells JCK, Eaton S, Fewtrell M. A Breastfeeding Relaxation Intervention Promotes Growth in Late Preterm and Early Term Infants: Results from a Randomized Controlled Trial. *Nutrients*. 2022;14(23):5041. doi:10.3390/nu14235041

5. Dib S, Wells JCK, Fewtrell M. Mother And late Preterm Lactation Study (MAPLeS): a randomised controlled trial testing the use of a breastfeeding meditation by mothers of late preterm infants on maternal psychological state, breast milk composition and volume, and infant behaviour and growth. *Trials*. 2020;21(1):318. doi:10.1186/s13063-020-4225-3

6. Feher SD, Berger LR, Johnson JD, Wilde JB. Increasing breast milk production for premature infants with relaxation/imagery audiotape. *Pediatrics*. 1989;83(1):57-60.

7. Keith DR, Weaver BS, Vogel RL. The Effect of Music-Based Listening Interventions on the Volume, Fat Content, and Caloric Content of Breast Milk–Produced by Mothers of Premature and Critically Ill Infants. *Advances in Neonatal Care*. 2012;12(2):112-119. doi:10.1097/ANC.0b013e31824d9842

8. Kittithanesuan Y, Chiarakul S, Kaewkungwal J, Poovorawan Y. Effect of music on immediately postpartum lactation by term mothers after giving birth: A randomized controlled trial. *Journal of the Medical Association of Thailand*. 2017;100(8):834-842. http://www.jmatonline.com/index.php/jmat/article/viewfile/7397/7383

9. Massa K, Ramireddy S, Ficenec S, Mank C, Josephsen J, Babbar S. A Randomized Control Trial of Meditation for Mothers Pumping Breastmilk for Preterm Infants. *Am J Perinatol*. Published online March 3, 2022. doi:10.1055/a-1787-7576

© 2024 Levene I et al. *JAMA Pediatrics.*

10. Mohd Shukri NH, Wells J, Eaton S, et al. Randomized controlled trial investigating the effects of a breastfeeding relaxation intervention on maternal psychological state, breast milk outcomes, and infant behavior and growth. *Am J Clin Nutr*. 2019;110(1):121-130. doi:10.1093/ajcn/nqz033

11. Shukri NHM, Wells J, Mukhtar F, Lee MHS, Fewtrell M. Study protocol: An investigation of mother-infant signalling during breastfeeding using a randomised trial to test the effectiveness of breastfeeding relaxation therapy on maternal psychological state, breast milk production and infant behaviour and growt. *Int Breastfeed J*. 2017;12:33. doi:https://dx.doi.org/10.1186/s13006-017-0124-y

12. Perez-Blasco J, Viguer P, Rodrigo MF. Effects of a mindfulness-based intervention on psychological distress, well-being, and maternal self-efficacy in breast-feeding mothers: results of a pilot study. *Arch Womens Ment Health*. 2013;16(3):227-236. doi:10.1007/s00737-013-0337-z

13. Ramesh B, Sundar S, Ghose S, D G, B S, Ezhumalai G. Evaluating the effect of music therapy on the establishment of lactogenesis and maternal breastfeeding satisfaction levels. *International Journal of Medical and Health Research*. 2020;6(6):5-9.

14. SefidHaji S, Aziznejadroshan P, Mojaveri MH, Nikbakht HA, Qujeq D, Amiri SRJ. Effect of lullaby on volume, fat, total protein and albumin concentration of breast milk in premature infants' mothers admitted to NICU: a randomized controlled trial. *Int Breastfeed J*. 2022;17(1):71. doi:10.1186/s13006-022-00511-7

15. Shabnam J, Mahsa A, Manoochehr M, Sonia O. Effect of music on the growth monitoring of low birth weight newborns. *Int J Afr Nurs Sci*. 2021;14:100312. doi:10.1016/j.ijans.2021.100312

16. Varişoğlu Y, Güngör Satilmiş I. The Effects of Listening to Music on Breast Milk Production by Mothers of Premature Newborns in the Neonatal Intensive Care Unit: A Randomized Controlled Study. *Breastfeeding Medicine*. 2020;15(7):465-470. doi:10.1089/bfm.2020.0027

17. Yu J, Wells J, Wei Z, Fewtrell M. Randomized trial comparing the physiological and psychological effects of different relaxation interventions in Chinese women breastfeeding their healthy term infant. *Breastfeed Med*. 2019;14(1):33-38. doi:10.1089/bfm.2018.0148

18. Yu J, Wei Z, Wells JCK, Fewtrell M. Effects of relaxation therapy on maternal psychological status and infant growth following late preterm and early-term delivery: a randomized controlled trial. *Am J Clin Nutr*. 2023;117(2):340-349. doi:10.1016/j.ajcnut.2022.12.002

19. Yu J, Wells J, Wei Z, Fewtrell M. Effects of relaxation therapy on maternal psychological state, infant growth and gut microbiome: protocol for a randomised controlled trial investigating mother-infant signalling during lactation following late preterm and early term delivery. *Int Breastfeed J*. 2019;14(1):50. doi:10.1186/s13006-019-0246-5

© 2024 Levene I et al. *JAMA Pediatrics.*
