# Supplementary material for: Relaxation Therapy and Human Milk Feeding Outcomes: A Systematic Review and Meta-Analysis
Source: JAMA Pediatr. 2024 May 6;178(6):567–76. doi: 10.1001/jamapediatrics.2024.0814 (PMC11074933; doi:10.1001/jamapediatrics.2024.0814)
Supplement: Supplement 2. — Data Sharing Statement. [file jamapediatr-e240814-s002.pdf]

Data Sharing Statement

Levene. Relaxation Therapy and Human Milk Feeding Outcomes. *JAMA Pediatr*. Published May 06, 2024. doi:10.1001/jamapediatrics.2024.0814

Data
Data available: Yes
Data types: Other (please specify)
Additional Information: Included studies have responsibility for their own data. Records of individual data extraction and risk of bias assessment are available on request to the corresponding author.
How to access data: On request to the corresponding author
When available: With publication

Supporting Documents
Document types: None

Additional Information
Who can access the data: Anyone requesting the data
Types of analyses: Any purpose
Mechanisms of data availability: Without investigator support
